# *In silico* study of the mechanisms of hypoxia and contractile dysfunction during ischemia and reperfusion of hiPSC cardiomyocytes

**DOI:** 10.1242/dmm.050365

**Published:** 2024-04-26

**Authors:** Mohamadamin Forouzandehmehr, Michelangelo Paci, Jari Hyttinen, Jussi T. Koivumäki

**Affiliations:** ^1^ Faculty of Medicine and Health Technology, Tampere University, 33520 Tampere, Finland; ^2^ Department of Electrical, Electronic, and Information Engineering ‘Guglielmo Marconi’, University of Bologna, 47522 Cesena, Italy

**Keywords:** *In silico* modeling, Human stem cell-derived cardiomyocytes, Action potential, Levosimendan, Cardiac metabolism, Ischemia, Reperfusion, Pharmacology

## Abstract

Interconnected mechanisms of ischemia and reperfusion (IR) has increased the interest in IR *in vitro* experiments using human induced pluripotent stem cell-derived cardiomyocytes (hiPSC-CMs). We developed a whole-cell computational model of hiPSC-CMs including the electromechanics, a metabolite-sensitive sarcoplasmic reticulum Ca^2+^-ATPase (SERCA) and an oxygen dynamics formulation to investigate IR mechanisms. Moreover, we simulated the effect and action mechanism of levosimendan, which recently showed promising anti-arrhythmic effects in hiPSC-CMs in hypoxia. The model was validated using hiPSC-CM and *in vitro* animal data. The role of SERCA in causing relaxation dysfunction in IR was anticipated to be comparable to its function in sepsis-induced heart failure. Drug simulations showed that levosimendan counteracts the relaxation dysfunction by utilizing a particular Ca^2+^-sensitizing mechanism involving Ca^2+^-bound troponin C and Ca^2+^ flux to the myofilament, rather than inhibiting SERCA phosphorylation. The model demonstrates extensive characterization and promise for drug development, making it suitable for evaluating IR therapy strategies based on the changing levels of cardiac metabolites, oxygen and molecular pathways.

## Teaser

**Summary:** A computer model of stem-cell derived cardiomyocytes was used to map the subcellular electro-mechano-energetics onto the mechanism of action of levosimendan, the oxygen consumption rate and relaxation dysfunction in ischemia and reperfusion.

## INTRODUCTION

Ischemic heart disease (IHD), resulting from an imbalance in the supply and demand of oxygen and nutrients to the heart, is the leading cause of mortality across the globe (Nowbar et al., 2019). The majority of the experiments on ischemia and ischemia reperfusion (IR) have been done with animal models, whereas human-based investigations are not very common (Dutta et al., 2017; Gaballah et al., 2022). Aside from ethical concerns, the extrapolation of mechanisms and insights from animal data is challenging. Overcoming these hurdles, human induced pluripotent stem cell-derived cardiomyocytes (hiPSC-CMs) offer promising opportunities for investigating the pathogenesis and mechanisms of action of pharmaceutical compounds in a human-based model (Forouzandehmehr et al., 2022; Gaballah et al., 2022). Of importance, hiPSC-CMs have been shown to be a dependable approach for identifying the pro-arrhythmic effects of medications (Selli et al., 2023). Furthermore, organ-on-chip approaches offer novel measurement modalities and robust ways to control the measurement conditions, such as precise local regulation of oxygen levels (Gaballah et al., 2022).

The combination of hiPSC-CM-based *in vitro* experiments and computational models of human adult ventricular cardiomyocytes (hV-CMs) has been introduced as a capable alternative for the hERG-based QT method, which utilizes hERG channel activity to assess the QT interval in the heart, in safety cardiac pharmacology and preclinical assessments (Paci et al., 2015). The updated methodology has been acknowledged by the pharmaceutical industry and, congruently, the US Food and Drug Administration (FDA) has supported this, i.e. the comprehensive *in vitro* proarrhythmia assay (Sager et al., 2014), as the paradigm for the assessment of new molecular entities.

Preserving cardiac functionality and preventing IR-induced arrhythmias are imperative principles in the management of IHD. Levosimendan (LEVO), a known calcium (Ca^2+^) sensitizer with inotropic effects, is in clinical use for heart failure treatment (Kurt, 2009). Recently, the antiarrhythmic effect of LEVO was reported by Gaballah et al. (2022), who used an hiPSC-CM-based IHD-on-a-chip single cell line model to investigate hypoxia-induced abnormalities of intracellular Ca^2+^ handling. However, the roles of ischemic action potential (AP) morphology, the subcellular and ionic interactions, and the electro-mechano-energetics in shaping the fate of cardiomyocytes in IR remain to be thoroughly addressed. Similarly, the mechanism of suppressing ventricular tachyarrhythmias by antiarrhythmic compounds is not entirely understood (Franz et al., 2014). Finally, to the best of our knowledge, no studies have quantitatively investigated the electro-mechano-energetic coupling and communications of the myofilament and sarcoplasmic reticulum Ca^2+^-ATPase (SERCA) metabolism to IR and IR-induced arrhythmia in cardiomyocytes. Specifically, accounting for the metabolite sensitivity aspect in SERCA and contractile element (CE) calibration can potentially reveal the links between different mechanisms of IR, drug-induced effects and the electro-mechano-energetic crosstalk in IR in response to Ca^2+^-sensitizing drugs.

The emerging hiPSC-CM *in vitro* data obtained in oxygen-controlled settings (Gaballah et al., 2022; Häkli et al., 2022) enable computational approaches for investigating IR, thus taking a step toward filling the aforementioned gaps. Therefore, in this work, we present an *in silico* hiPSC-CM model that includes metabolite sensitivity in the CE and SERCA pump (hereafter hiMCES model), building upon our previous studies (Forouzandehmehr et al., 2021b, 2022). We introduce a model of oxygen (O_2_) dynamics linking the cellular ionic and CE ATPase rate changes to extracellular O_2_ concentration. The presented computational model was validated against hiPSC-CM and animal experiment data; specifically, the core of ischemic, IR and LEVO effects were validated against *in vitro* hiPSC-CM data (Gaballah et al., 2022; Häkli et al., 2021, 2022). Firstly, we used this model to investigate the impaired electro-mechano-energetic coupling in IR and portray the subcellular signature unique to IR. Secondly, we elucidated the cardioprotective effect of LEVO on the Ca^2+^ and AP abnormalities in IR and quantified its influence on the subcellular mechanisms contributing to IR.

## RESULTS

### Characteristics of the hiMCES model in control conditions

First, AP, Ca^2+^ transient (CaT) and contractile biomarkers were computed to evaluate the hiMCES model in the control condition regarding the experimental data ranges (Table S1). The increased energetic detail did not significantly alter the model readouts in the control condition, and the hiMCES model successfully recapitulated all the listed biomarkers within the experimental ranges. Of note, the lower maximum upstroke velocity (dV/dt_max_) simulated by the hiMCES model in comparison with its predecessors (Table S1) is due to a smaller simulated fast Na^+^ current (I_Na_) magnitude. In Fig. S17, we have given the factors that contribute to change in I_Na_ and ultimately dV/dt_max_ based on the electrophysiology part that hiMCES inherits from the Paci2020 model (Paci et al., 2020). Briefly, the two new elements in hiMCES model, SERCA pump flux (I_up_) and an ATP-sensitive K^+^ current (I_KATP_), affect the ionic currents and consequently the membrane potential V_m_ in the order illustrated by arrows in Fig. S17, in accord with the electrophysiology of the model equations in Paci et al. (2018, 2020). Change in V_m_ results in change in the fast Na^+^ current I_Na_ and, ultimately, the maximum upstroke velocity dV/dt_max_. Correspondingly, Fig. S1 shows the main traces of the hiMCES model in the control condition in comparison to the hiPSC-CM models previously developed in our laboratory and hiPSC-CM experimental data (Pioner et al., 2020; Ruan et al., 2016).

### Simulation of ischemic conditions

During IR, the SERCA submodel is subject to metabolite changes that drastically affect the Ca^2+^ sensitivity and the amplitude of Ca^2+^ flux to the sarcoplasmic reticulum (SR) (Fig. S2). The hiMCES model with the original SERCA submodel failed to develop AP and AP/CaT abnormalities during IR. To find a unique set of parameters for the SERCA-rendering hiMCES model to generate AP and capture AP/CaT abnormalities, we reparametrized the SERCA model (Table S2) based on the sensitivity tests on the amplitude of SERCA pump rate and Ca^2+^ sensitivity (Figs S3-S10). Additionally, Fig. S11 shows the Ca^2+^ sensitivity of SERCA pump rates at different pH levels and confirms that the calibrated SERCA pump for ischemic simulations mimics the acidic response in previously reported data (Ji et al., 1999; Tran et al., 2009).

To elaborate, we found the set of parameters through an informed manual tuning process. In brief, we used, e.g. 10, 20 and 50% rises starting with a first guess in a range of 0.1 to 5 times the baseline values. Within the assumed boundaries (0.1 to 5 times the baseline values), the optimization goal was reaching a maximum pump rate while overall keeping the pump rate versus the negative logarithm of the Ca^2+^ concentration (pCa) curve in the acidic zone (Fig. S11).

**Fig. 1.**
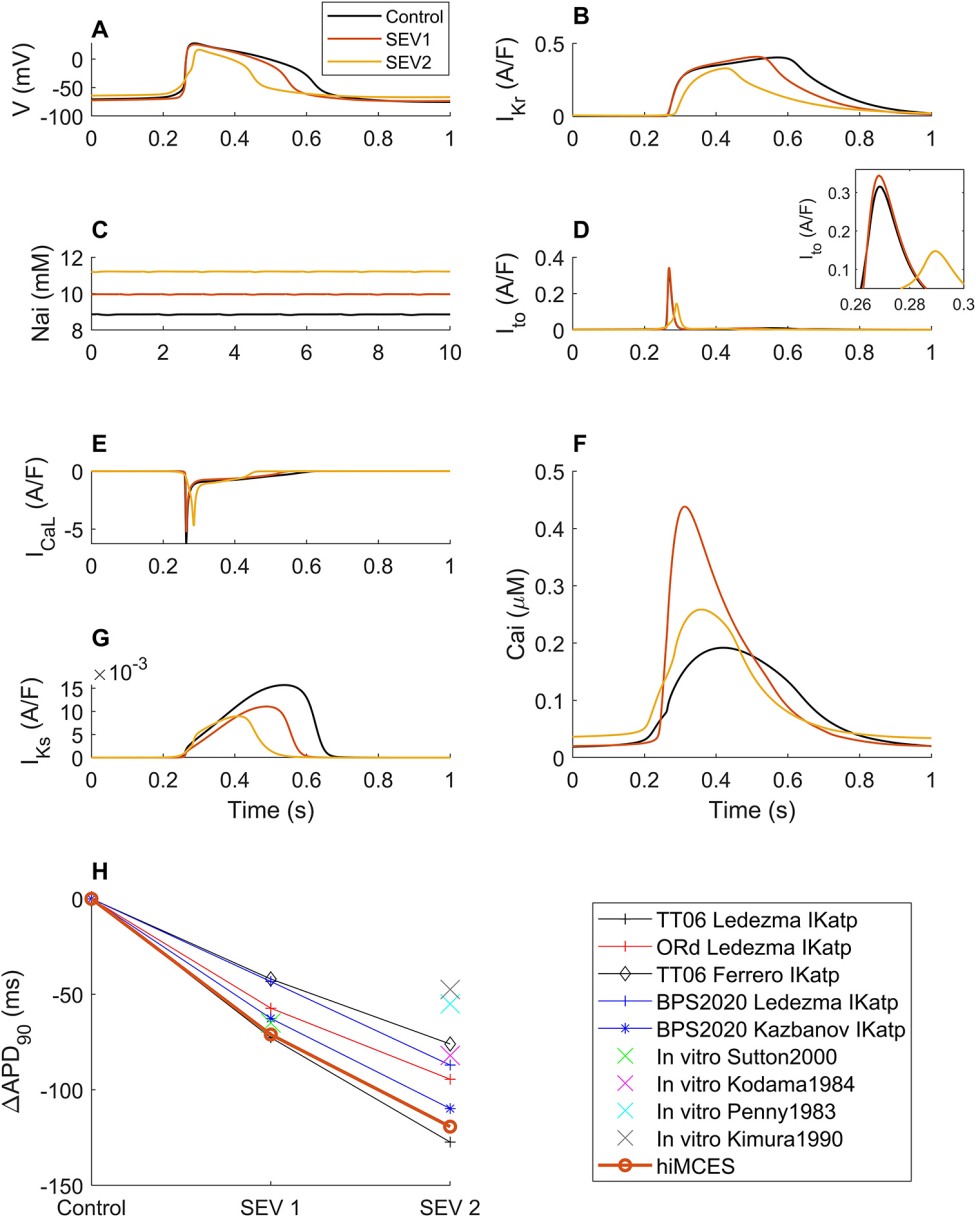
**Response of the hiMCES model to different severities of ischemia.** (A) Action potentials (AP or V). (B) Rapid delayed rectifier K^+^ current (I_Kr_). (C) Intracellular Na^+^ concentration (Na_i_). (D) Transient outward K^+^ current (I_to_). (E) L-type Ca^2+^ current (I_CaL_). (F) Ca^2+^ transients (Ca_i_). (G) Slow delayed rectifier K^+^ current (I_Ks_). (H) AP duration alterations at 90% of repolarization (ΔAPD_90_). Listed computational data: TT06 Ledezma (Ledezma et al., 2019), ORd (Ledezma et al., 2019), TT06 Ferrero (Weiss et al., 2009) and BPS2020 (Forouzandehmehr et al., 2021a). Listed *in vitro* data: Sutton2000 (Sutton, 2000), Kodama1984 (Kodama et al., 1984), Penny1983 (Penny and Sheridan, 1983) and Kimura1990 (Kimura et al., 1990).

We evaluated the model results in two ischemic severities (SEV1 and SEV2) as given in Fig. 1. The AP durations decreased and the depolarization time increased in response to ischemic conditions at two severities (Fig. 1A) consistently with data of hiPSC-CMs subjected to hypoxia *in vitro* (Häkli et al., 2021, 2022). The predicted reduction in potassium currents (Fig. 1B,G) contributes to increased (less negative) resting membrane voltage consistently with hiPSC-CM data (Deo et al., 2020; Doss et al., 2012). Moreover, the simulated increase in maximum diastolic potential at SEV2 was 11%, which is in the maximum diastolic potential increase range (0-31.5%) reported from *in vitro* data of hiPSC-CMs in ischemia (Davis et al., 2021). Furthermore, the model identifies a significant reduction in transient outward K^+^ current (I_to_) (Fig. 1D, SEV2) as a major contributor to the repolarization phase of AP in hiPSC-CMs (Koivumäki et al., 2018). Our model also confirms the direct correlation of L-type Ca^2+^ current (I_CaL_) with AP plateau height (Fig. 1A,E) reported experimentally in ischemia (Rogers et al., 2021). The increased intracellular Na^+^ concentration (Fig. 1C) in ischemic conditions is in line with reported hiPSC-CM data in hypoxic conditions (Gaballah et al., 2022). At SEV2, our model simulated an elevation of the CaT baseline in accord with the significant increase in the diastolic Ca^2+^ concentration ([Ca^2+^]) reported for isolated rat and rabbit hearts in ischemic conditions (Eberli et al., 2000) (Fig. 1F). At SEV2, the model simulated 17.4% reduction in decay time from 90 to 10% (tDecay_90,10_) of CaT peak (317 to 262 ms) compared with the control condition, which is quantitatively in agreement with the 15.2-36.3% reduction experimentally reported for hiPSC-CMs after 4 h of hypoxia (Gaballah et al., 2022). Notably, the reverse mode of Na^+^/Ca^2+^ exchanger (NCX) was augmented (∼8%) in ischemia (Fig. S16), which also contributes to CaT elevation, and this is in accord with the data reported for Langendorff-perfused mice hearts (Imahashi et al., 2005) in ischemic conditions.

Finally, we simulated and compared the changes in AP duration alterations at 90% of repolarization (APD_90_) with *in vitro* data obtained from human cardiomyocytes (Sutton, 2000) (after 3 min), cat (Kimura et al., 1990) (after 10-15 min) and guinea pig (Penny and Sheridan, 1983) (after 10-15 min) as given in Fig. 1H. The changes in APD_90_ were also compared against other computational models of ischemia and are summarized in Fig. 1H. For example, the ‘ten Tusscher 2006’ (TT06) hV-CM model (Ten Tusscher and Panfilov, 2006) was used by Weiss et al. (2009) in an ischemic study (5 and 10 min severities), adopting the I_KATP_ formulation from Ferrero et al. (1996). As Fig. 1H details, the APD_90_ changes simulated by hiMCES qualitatively agree with the observed trend.

We also simulated the effect of different mechanisms of ischemia, i.e. hyperkalemia, acidosis and hypoxia, separately (Fig. S14). The increase in ryanodine receptor (RyR)-sensitive release current (I_rel_) in ischemia (Fig. S14F) is consistent with a previous computational investigation using the metabolite-sensitive SERCA pump (Tran et al., 2009) integrated into the ORd (O'Hara et al., 2011) hV-CM model (Liu et al., 2019).

Finally, based on the introduced formulation capturing the model oxygen dynamics (Eq. 12), we simulated the effect of ischemia as a whole at SEV1 and SEV2 (Fig. S12A,B), and separately by mechanism (Fig. S12C). The model predicted reductions in the amplitude of oxygen consumption rate (OCR) at SEV1 and SEV2 ischemia at the steady state. Importantly, the simulated increase of OCR, modeled by dO_2_/dt, at the onset of ischemia (Fig. S12D) is in accord with myocardial oxygen studies on patients with IHD (Deedwania and Carbajal, 1992; Laslett et al., 1985).

### LEVO simulations

**Fig. 2.**
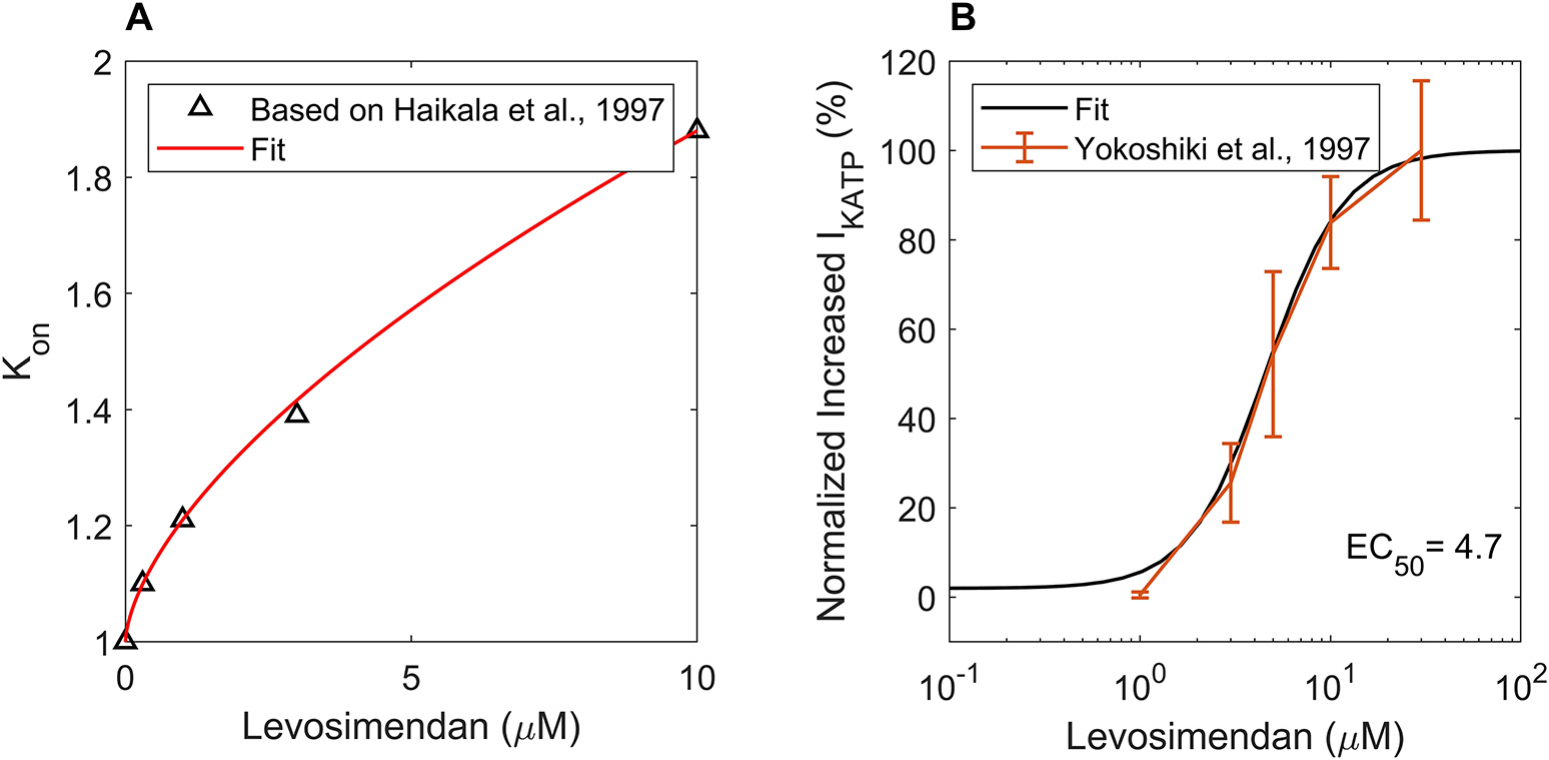
**The hiMCES model incorporates the role of myofilament Ca^2+^-binding affinity and the ATP-sensitive K^+^ channel in modeling the mechanism of action of LEVO.** (A) The computed K_on_ scaling coefficients calculated for different concentrations of levosimendan (LEVO) based on Haikala (1997). (B) Normalized increased I_KATP_ due to different doses of LEVO from Yokoshiki et al. (1997a).

Using the IC_50_ values and Hill coefficients of LEVO (Passini et al., 2019) in the channel-blocking method, we calculated the current inhibitions as given in Table S3. Furthermore, LEVO is an inotropic Ca^2+^ sensitizer; one of its mechanisms of action is increasing troponin C affinity for Ca^2+^ and stabilizing the conformation of troponin C (Gaballah et al., 2022). Regarding the dose-dependent LEVO-induced contraction data by Haikala (1997), we calculated the values of K_on_ scaling coefficients corresponding to the simulated developed tensions by the hiMCES model (Fig. 2A); K_on_ is the parameter regulating the transition between the unbound to the Ca^2+^-bound troponin state (Rice et al., 2008) (Fig. 6). Finally, the effect of LEVO as an I_KATP_ activator (Gaballah et al., 2022; Parissis et al., 2007; Pathak et al., 2013) was also considered in this study. We found corresponding metabolite-sensitive scaling parameter (f_KATP_) values, resulting in the increased currents observed (Fig. 2B) for the studied doses of LEVO based on data from Yokoshiki et al. (1997a). We selected 0.3, 2 and 10 µM doses of LEVO in the simulations, to be consistent with the LEVO animal experiment research (Edes et al., 1995; Yokoshiki et al., 1997a) and hiPSC-CM research (Selli et al., 2023). For the f_KATP_ activation, we followed data presented in fig. 3B of Yokoshiki et al. (1997a) and normalized the current increase with respect to the maximal stimulatory effect (E_max_)=46.8 A/F. Specifically, the 84% current increase in Table S3 is also consistent and within the range of increased I_KATP_ due to 10 µM LEVO (60.1 to 386.6%) reported in Yokoshiki et al. (1997b) at 0 mV.

**Fig. 3.**
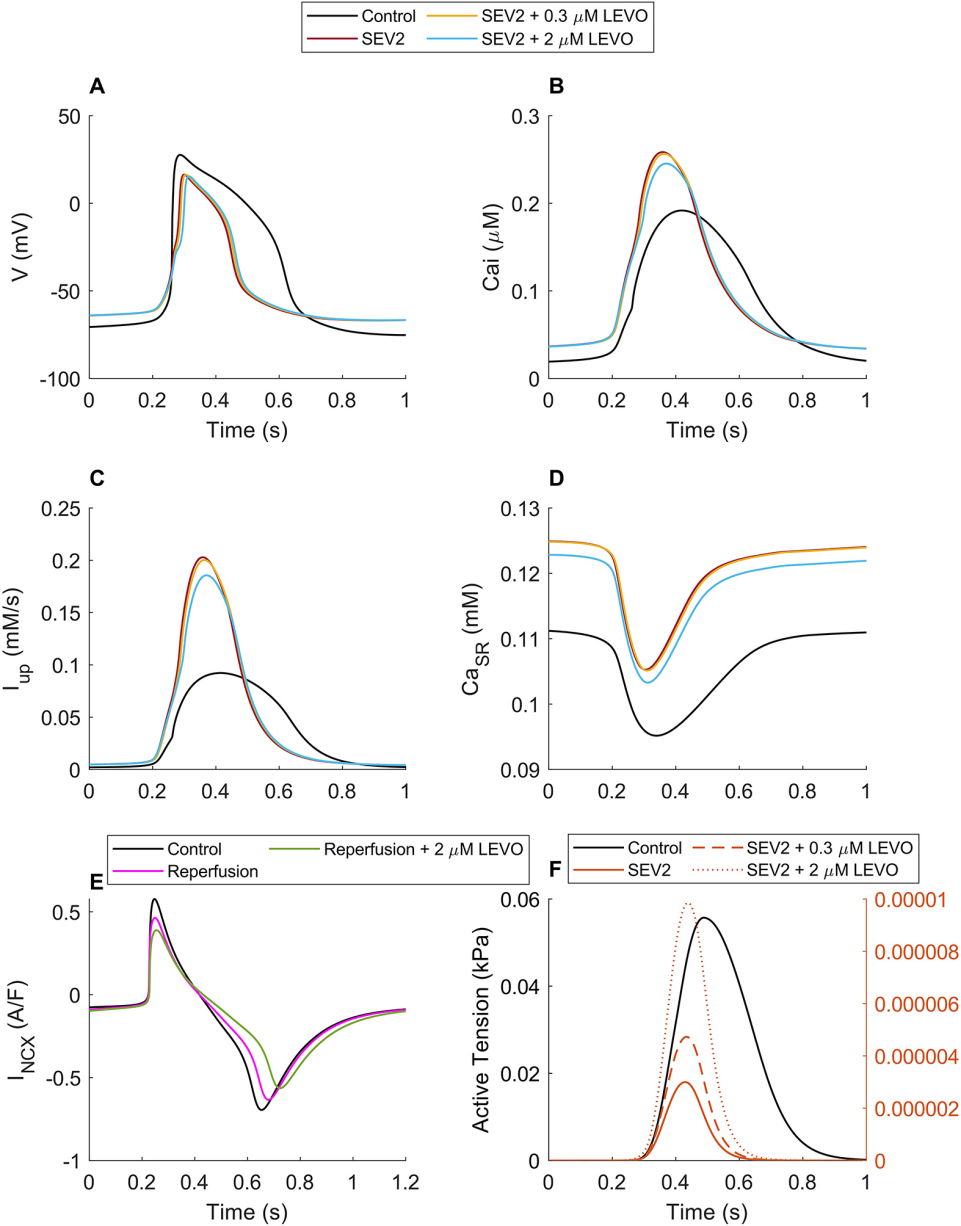
**The response of the hiMCES model in ischemia reperfusion to different concentrations of LEVO.** (A) Action potentials (V). (B) Ca^2+^ transients (Ca_i_). (C) Ca^2+^ uptake by SERCA (I_up_). (D) Sarcoplasmic reticulum Ca^2+^ concentration (Ca_SR_). (E) Na^+^-Ca^2+^ exchanger current (I_NCX_). (F) Active tensions.

We evaluated the effect of 0.3 and 2 µM of LEVO on SEV2 ischemia in the hiMCES model (Fig. 3). LEVO had a marginal prolonging effect on the AP repolarization phase (Fig. 3A; Table S4), in accord with a recent *in vitro* hiPSC-CM experiment (Selli et al., 2023), and reported dose-dependent LEVO association with the prolongation of QT interval in healthy individuals and patients with heart failure (Ng, 2004). At 0.3 and 2 µM concentrations, LEVO did not significantly alter the CaT values (Fig. 3B), yet it resulted in a significant increase in the active tension (Fig. 3F), consistent with the Ca^2+^-sensitizing and inotropic characteristics of LEVO (Gaballah et al., 2022; Ng, 2004). The model also predicted a significant increase in contractile ATPase rate in response to LEVO (Fig. S15I). As Fig. 3C,D shows, 2 µM LEVO reduced the Ca^2+^ flux via SERCA pumps and the SR Ca^2+^ concentration by ∼8.4% and ∼1.9%, respectively, moving them toward more physiological levels. The increase in simulated tensions at SEV1 and SEV2 due to 0.3 µM of LEVO were 25% and 60%, respectively. This is quantitatively comparable with the increase in developed tension range of 14.6 to 54.3% reported experimentally for ischemic human myocardium at 37°C (Soeding et al., 2011). Furthermore, the predicted LEVO-induced decrease in Na^+^-Ca^2+^ exchanger current (I_NCX_) (Fig. 3E) in reperfusion is in line with the effect of LEVO on NCX in cultured human cardiomyocyte progenitor cell-derived cardiomyocytes experiencing anoxia reoxygenation (Li et al., 2014). Our model simulated ∼10% reduction in NCX reverse mode due to 2 µM of LEVO. In comparison, Li et al. (2014) reported 44.5 to 85.0% decrease of NCX reverse mode due to 2 µM of LEVO. We discuss the possible responsible factors for this difference in the Discussion. The effect of LEVO on the fractional cell shortening (Fig. S15J) agrees with the *in vitro* data from rat cardiomyocytes in heart failure (Louhelainen et al., 2007).

### Ischemia reperfusion and LEVO effects

As the results show, the hiMCES IR model could capture ischemic-induced arrhythmia, in early afterdepolarization (EAD) format, and CaT abnormalities (Fig. S13C,D) consistently with the experimental data of hiPSC-CMs in hypoxia (Gaballah et al., 2022). Specifically, the predicted CaT abnormalities in the form of high and low peaks and irregular double peaks in the plateau phase (Fig. S13D) were also observed experimentally in hiPSC-CMs in ischemia (Gaballah et al., 2022). Moreover, the calculated maximum upstroke velocity (dV/dt_max_) of AP in the ischemic phase, prior to the development of EADs, was reduced to around a quarter of the value in the control condition (34.9 to 9.6 V/s). The maximum upstroke velocity of APs inversely correlates with the depolarization time, and the simulated change resides within the reported increase in the depolarization time for *in vitro* hiPSC-CMs in IR (Häkli et al., 2022).

We also studied the effect of augmentation of the slow delayed rectifier K^+^ current (I_Ks_) on the model results in IR. As can be seen in Fig. S13E,F, enhanced I_Ks_ abolished the AP and CaT abnormalities consistently with the IR model of rabbit cardiomyocytes (Guo et al., 2012). This also resulted in abolishing the ischemic-induced contractile irregularities, in terms of aftercontractions, consequently (Fig. S13G). Aftercontractions in myocardial ischemia is reported to be a result of disturbed cellular diastolic Ca^2+^ transient and is associated with lethal ventricular arrhythmias (Prunier et al., 2008).

**Fig. 4.**
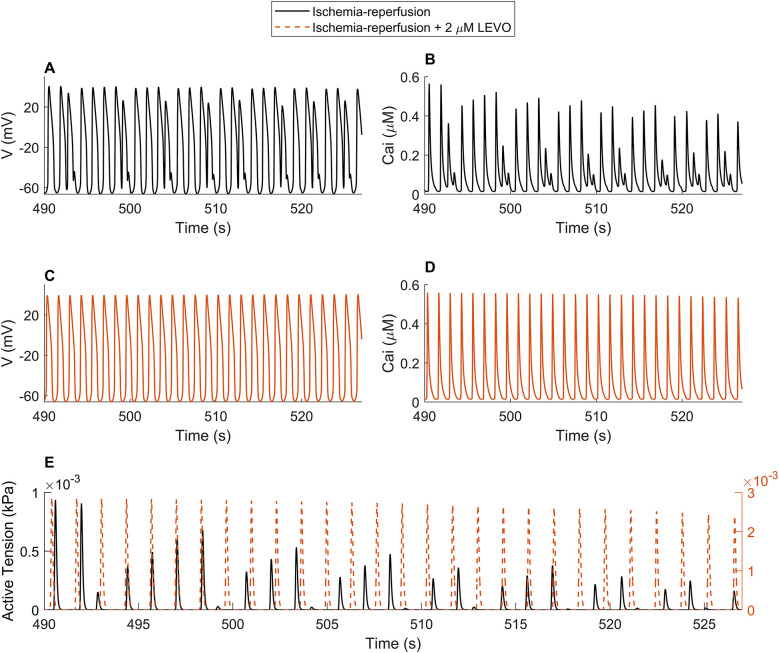
**Response of the hiMCES model to 2 µM LEVO in ischemia reperfusion simulations.** (A,B) Action potential (AP or V) and Ca^2+^ transients (CaTs, Ca_i_) in ischemia reperfusion (IR). (C,D) APs and CaTs in IR+2 µM LEVO. (E) Active tensions in IR and IR+2 µM LEVO.

Moreover, to understand the recently reported antiarrhythmic effect of LEVO (Gaballah et al., 2022), we investigated its effect at a 2 µM dose on AP, CaT and simulated active tensions developed in IR. Fig. 4A-D shows that the EADs were abolished and the CaT plateau abnormalities were substantially reduced, in agreement with the use of 2 µM LEVO on hypoxic hiPSC-CMs in an *in vitro* study (Gaballah et al., 2022). Naturally, this led to a favorable effect on contractility due to abolishment of aftercontractions (Fig. 4E).

**Fig. 5.**
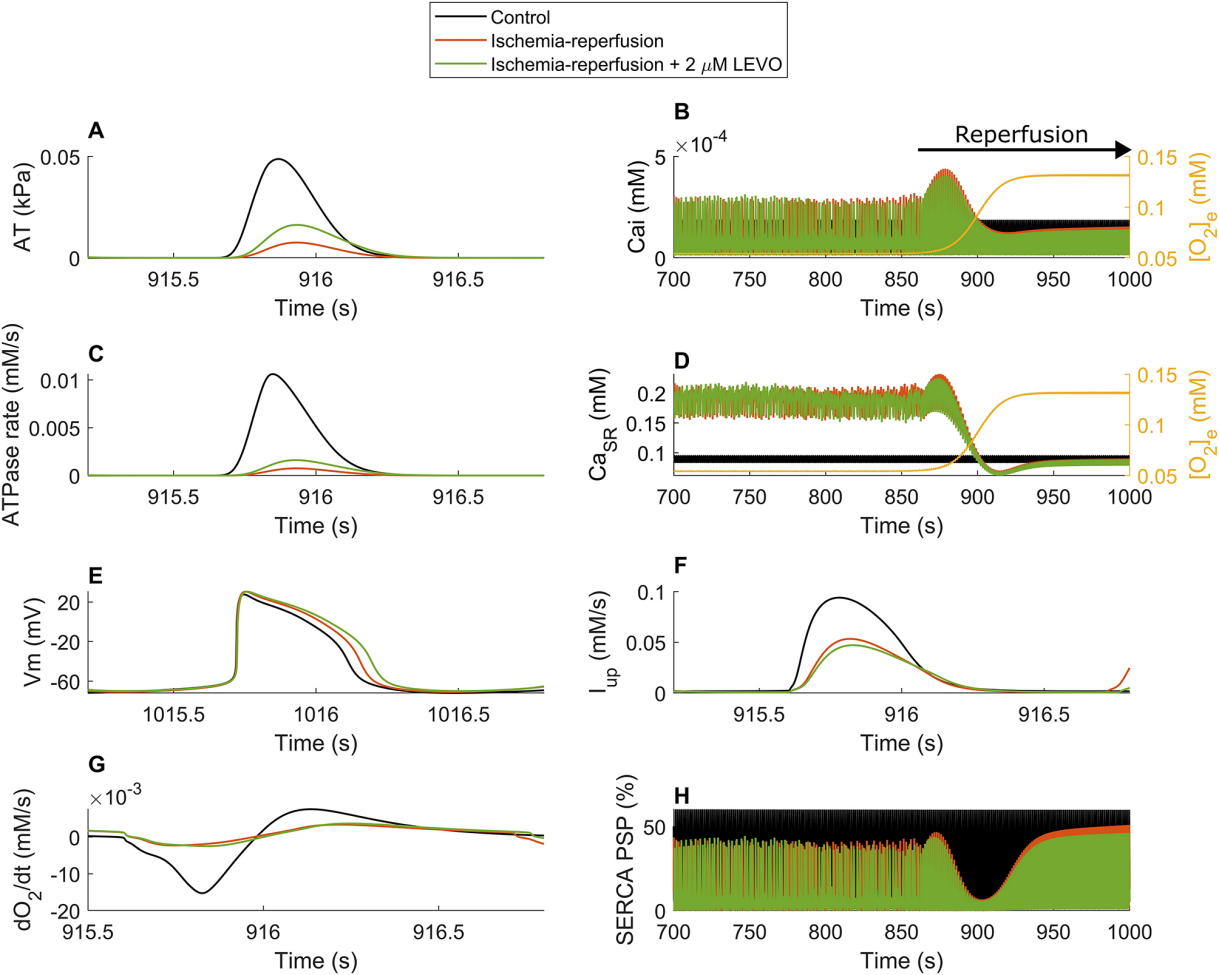
**The ischemia reperfusion hiMCES model subjected to 2 µM LEVO.** (A) Active tensions (AT). (B) Ca^2+^ transients (Ca_i_). (C) Contractile ATPase rate. (D) Sarcoplasmic reticulum Ca^2+^ concentrations (Ca_SR_). (E) Action potentials shown 50 s postreperfusion transition (V_m_). (F) Ca^2+^ uptake flux of SERCA pump (I_up_). (G) Oxygen consumption rate (dO_2_/dt). (H) Percentage of SERCA phosphorylation (PSP).

Lastly, we show the model response to 2 µM LEVO during and after the reperfusion transition in Fig. 5. The model predicted an upsurge in the active tension (Fig. 5A) and contractile ATPase rate (Fig. 5C) in response to LEVO, despite the general reduction of CaT at late reperfusion phase (Fig. 5B). Correspondingly, an insignificant OCR change due to LEVO was also observed in the reperfusion and postreperfusion phases (Fig. 5G), consistent with the reported Ca^2+^ sensitization effect in ischemia in human studies (Ng, 2004). Taken together with the changes in active tension and ATPase rate (Fig. 5A,C), an enhancement in the cardiac energy consumption due to LEVO is implied, which is similar to its effect during the ischemic phase (Fig. S15G). This favorable energetic outcome is consistent with the restorative effect of LEVO on the contractility and altered cardiac energetics in human subjects (Ng, 2004), hiPSC-CMs (Gaballah et al., 2022) and Langendorff guinea pig hearts (Haikala, 1997) in ischemia. The IR-LEVO simulation further predicts a biphasic trend in CaT and Ca^2+^ concentration in the SR (CaSR) starting from the onset of reperfusion (Fig. 5B,D) and a significant reduction period of SERCA phosphorylation (Fig. 5H). Markedly, LEVO did not facilitate the return of CaT and CaSR to physoxia (Fig. 5B,D), nor of the AP profile, which is shown 50 s postreperfusion transition phase (Fig. 5E). Finally, a slight reduction of the impaired SERCA uptake pump due to 2 µM LEVO was predicted in the simulation results (Fig. 5F).

## DISCUSSION

### Model extension and characteristics

Myocardial infarction represents the first cause of mortality worldwide (Nowbar et al., 2019). Optimizing the treatments leading to restoration of physiological cardiomyocyte homeostasis and metabolism post-ischemic insult is of high importance (He et al., 2022). The pathophysiological mechanisms contributing to IR injuries such as disturbed cytosolic Ca^2+^ transient, oxidative stress, quick pH correction and phosphate overload (He et al., 2022) are intertwined and complex, necessitating in-depth quantitative investigations. To this end, it is crucial to develop computational techniques that match *in vitro* conditions and in-depth biophysical *in silico* models of electro-mechano-energetics at the cellular level.

To investigate IR pathophysiological mechanisms and drug effects, our first aim was to extend our previous *in silico* hiPSC-CM model (Forouzandehmehr et al., 2022) by adding a metabolic model of the SERCA pump and an oxygen dynamics model linking the cellular energetics to extracellular oxygen concentration ([O_2_]_e_). Of note, the introduced dynamic oxygen formulation takes the role of contraction ATPase rate into account as we anticipated that the significance of such inclusion would be even higher in studying IR (Bo et al., 2021; Laslett et al., 1985; McDougal and Dewey, 2017). We did not include I_KATP_ in the oxygen dynamic formulation as its impact on myocardial oxygen consumption rate has been reported insignificant in IR (Offstad et al., 1994).

### IR and LEVO

We surpassed the orthodox IC_50_-based inhibition formalism by considering I_KATP_ and troponin C affinity for the Ca^2+^-enhancing mechanism of LEVO (Gaballah et al., 2022). In excellent accordance with *in vitro* hiPSC-CM experiments in hypoxia (Gaballah et al., 2022), our model captured the substantial reduction of ischemia-induced AP and CaT abnormalities due to 2 µM LEVO. This could be associated with the effect of LEVO on the contributors to the repolarization reserve of the hiMCES model (Fig. S15A-C), which is congruent with QT interval prolongation in healthy subjects and patients with heart failure in response to LEVO (Ng, 2004).

Interestingly, our model predicted a change in the NCX profile by simulating a steep forward mode preceding the reverse mode at SEV1 (Fig. S15D). In contrast, for SEV2 ischemia, which corresponds to a more severe and prolonged hypoxic condition, our model simulated a more standard NCX profile (diminishing first steep forward mode) with an activated reverse mode (∼8%) (Fig. S16).

Our model simulated ∼10% reduction in NCX reverse mode in response to 2 µM of LEVO in reperfusion (Fig. 3E). The NCX reverse mode was calculated as the area under the curve (Fig. 3E). In comparison, Li et al. (2014) reported 44.5 to 85.0% decrease of NCX reverse mode due to 2 µM of LEVO in cardiomyocyte progenitor cell-derived cardiomyocytes undergoing reoxygenation. We believe that one of the factors responsible for this difference might be the *in vitro* protocol in Li et al. (2014), as it does not specifically take into account the role of L-type Ca^2+^ channel, SERCA and sarcolemmal Ca^2+^ ATPase pump current (I_pCa_) in how the NCX reverse mode based on the change in intracellular CaT dynamics is reported. Of note, the role of the aforementioned Ca^2+^ transporters has been proven to be significant in the developed CaT (Kernik et al., 2019)**.** Furthermore, the model predicted a significant increase in the Ca^2+^ flux to the myofilament due to LEVO at SEV1 ischemia (Fig. S15F). This indicates the potential of LEVO in mediating the transition of highly elevated CaT in ischemia and the underperformance of CaT due to the quick metabolite restoration in the reperfusion phase. In particular, it can be inferred from the simulations that LEVO can prolong the reperfusion transitions (Fig. 5). That finding has an interesting link to a recent computational study that suggested a two-step reperfusion protocol could minimize the cell damage in comparison to a rapid return to physoxia (Grass et al., 2022). Adding to this, our findings suggest that LEVO is a distinct inotrope that apparently does not augment the contractility at the expense of increased myocardial oxygen demand (Fig. 5G; Fig. S15G), which is consistent with previous analyses (Ng, 2004). Whether LEVO corrects the impaired contractile relaxation due to SERCA phosphorylation augmentation or other mechanisms was a question of research hypotheses before (Ng, 2004). Our model predictions suggest that the favorable effect of LEVO on correcting the impaired contractile and energetic relaxation is likely due to its Ca^2+^-binding, troponin C affinity-enhancing mechanism and intensifying Ca^2+^ flux to the myofilament rather than inhibition of SERCA phosphorylation that boosts removal of cytosolic Ca^2+^.

### Ischemia reperfusion and SERCA

As given in Tran et al. (2009) and simulated in Fig. S2, decreasing pH reduces the Ca^2+^ sensitivity of SERCA. We hypothesized that the reported increase in acidic residues and metabolic proton in IR (Grass et al., 2022) might affect SERCA function through a pathologic competitive proton binding. According to Tran et al. (2009), acidic residues impact proton and Ca^2+^ binding, and metabolic protons exhibit a higher affinity for Ca^2+^-binding sites in the SERCA luminal orientation and a lower affinity for SERCA-cytosol-facing Ca^2+^-binding sites. The ischemic acidic environment increases the influence of the reduction in the affinity of H^+^ binding to the luminal Ca^2+^-binding sites (K_d,Hsr_) in the reparameterization (Table S2). Therefore, increasing the gap between the affinity of H^+^ binding to the cytosol-facing Ca^2+^-binding sites (K_d,Hi_) and K_d,Hsr_ suggests that ischemia might intensify the competitive proton binding to the luminal-oriented Ca^2+^-binding sites of SERCA, thus elevating [Ca^2+^] in the SR as simulated in Figs 3D and 5D. Markedly, it further suggests that the upsurge in proton leak not only disrupts the cell metabolism, leading to elevation of oxidative stress in IR (Grass et al., 2022), but it also might worsen the disturbed pattern in proton binding, which results in a pathologic shift in SERCA Ca^2+^ sensitivity and pump function. Specifically, the calibration of the SERCA pump implied the inhibition of SERCA luminal Ca^2+^-binding sites as a potential therapeutic target that may facilitate attenuation of disordered competitive proton binding and thus prevent elevation of oxidative stress and elevated level of phosphates as the major mechanisms of IR injuries (He et al., 2022).

Of importance, our model predicts diastolic and relaxation dysfunction in CaT (Fig. 1F), contractile machinery (Fig. 3F; Fig. S15J) and energetics of the CE (Fig. S15I) in the ischemic condition. Previously, this contractile relaxation dysfunction was reported in heart-failure sepsis-induced mouse hearts, and it was suggested that this diastolic impairment is due to the inhibited SERCA phosphorylation (Joulin et al., 2009). Interestingly, our model also predicts a SERCA phosphorylation activity inhibition in the reperfusion phase (Fig. 5H). Markedly, in IR, this inhibition implies a mechanism contributing to the elevation of phosphate groups or amplification of the elevated phosphate groups that result in IR injuries (Ng, 2004).

Notably, the simulated increase in the OCR at the onset of ischemia (Fig. S12D) was caused by the higher rate of contraction ATPase in reaction to the shift in the CE metabolite concentrations from normal to low oxygen levels (Table 1). Regarding the treatment of IR, this further highlights the significance of using troponin C-associated Ca^2+^-sensitizing inotropes such as LEVO to restore heart contractile function without directly involving the oxygen consumption (Ng, 2004).

**Table 1.**
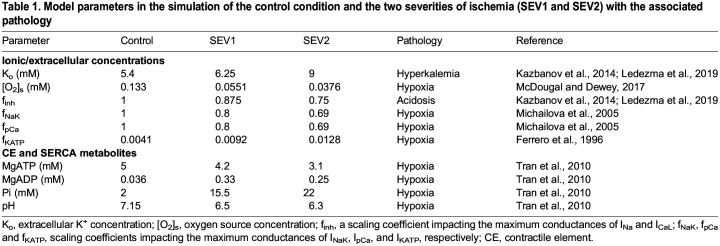
Model parameters in the simulation of the control condition and the two severities of ischemia (SEV1 and SEV2) with the associated pathology

### Limitations and future works

As a limitation, it should be noted that our model naturally inherits the mean-field approximation technique used to estimate the kinetics of cardiac crossbridge cycling in the CE from Rice et al. (2008) and Tran et al. (2010). Additionally, due to the very limited data availability on hiPSC-CM mitochondrial dynamics, our model also lacks this energetic component. Furthermore, the hiMCES model does not include an ATP-sensitive Na^+^-K^+^ pump current (I_NaK_) formulation; therefore, the reflection of I_NaK_ in OCR formulation is a simplification. Future works could also largely benefit from increasing crossbridge cycling details of the CE model toward deep-phenotyping the IR relaxation dysfunction and the mechanism of action of LEVO.

In this work, decoding the SERCA functionality in IR meets an in-depth *in silico* investigation of the effect of LEVO on hiPSC-CM electro-mechano-energetic coupling. Our findings suggest that there is a close mechanistic relationship between IR and sepsis-induced heart failure in terms of the role of SERCA role in diastolic dysfunction during mechanical relaxation and energetic function. This connection could be important for future drug development strategies. Furthermore, as a step toward identifying the link between the elevation of oxidative stress and Ca^2+^ overload as the two major mechanisms of IR injuries, our model suggests that the upsurge of proton leak in IR, which leads to elevated oxidative stress, also contributes to the ischemic Ca^2+^ overload through disrupted proton-binding kinetics in SERCA. LEVO simulations concluded that the drug-induced improvement on contractile relaxation dysfunction is due to its particular Ca^2+^-sensitizing mechanism, acting through Ca^2+^-bound troponin C and Ca^2+^ flux to the myofilament, rather than a secondary mechanism of SERCA phosphorylation inhibition. In conclusion, this study presented an innovative and strong computational framework for electro-mechano-energetic coupling in hiPSC-CMs, utilizing current advancements in obtaining human-based *in vitro* data. We demonstrated its potential in deep-phenotyping and pharmacological investigations in the context of ischemia and reperfusion.

## MATERIALS AND METHODS

### Integrating a metabolite-sensitive SERCA model

We previously developed an hiPSC-CM whole-cell model comprising a metabolite-sensitive CE, called hiMCE, to investigate mutation-specific hypertrophic cardiomyopathy (Forouzandehmehr et al., 2022). The hiMCE model consisted of a reparametrized model of metabolite-sensitive CE by Tran et al. (2017) integrated into the Paci2020 model of hiPSC-CM electrophysiology (Paci et al., 2020). To refine the cellular electro-mechano-energetic coupling in the model, here, we integrated a thermodynamic two-state model of the SERCA pump (Tran et al., 2009) to the hiMCE model and used this model, named hiMCES, to study the pathophysiology of IR, arrhythmia and the effect of LEVO (Simdax^®^, Orion Corporation). The choice of the two-state SERCA pump over the three-state pump was based on data in Tran et al. (2009) showing that the two models behave identical in the micromolar range of MgATP, which entails the working range of this study as well (Table 1).

**Fig. 6.**
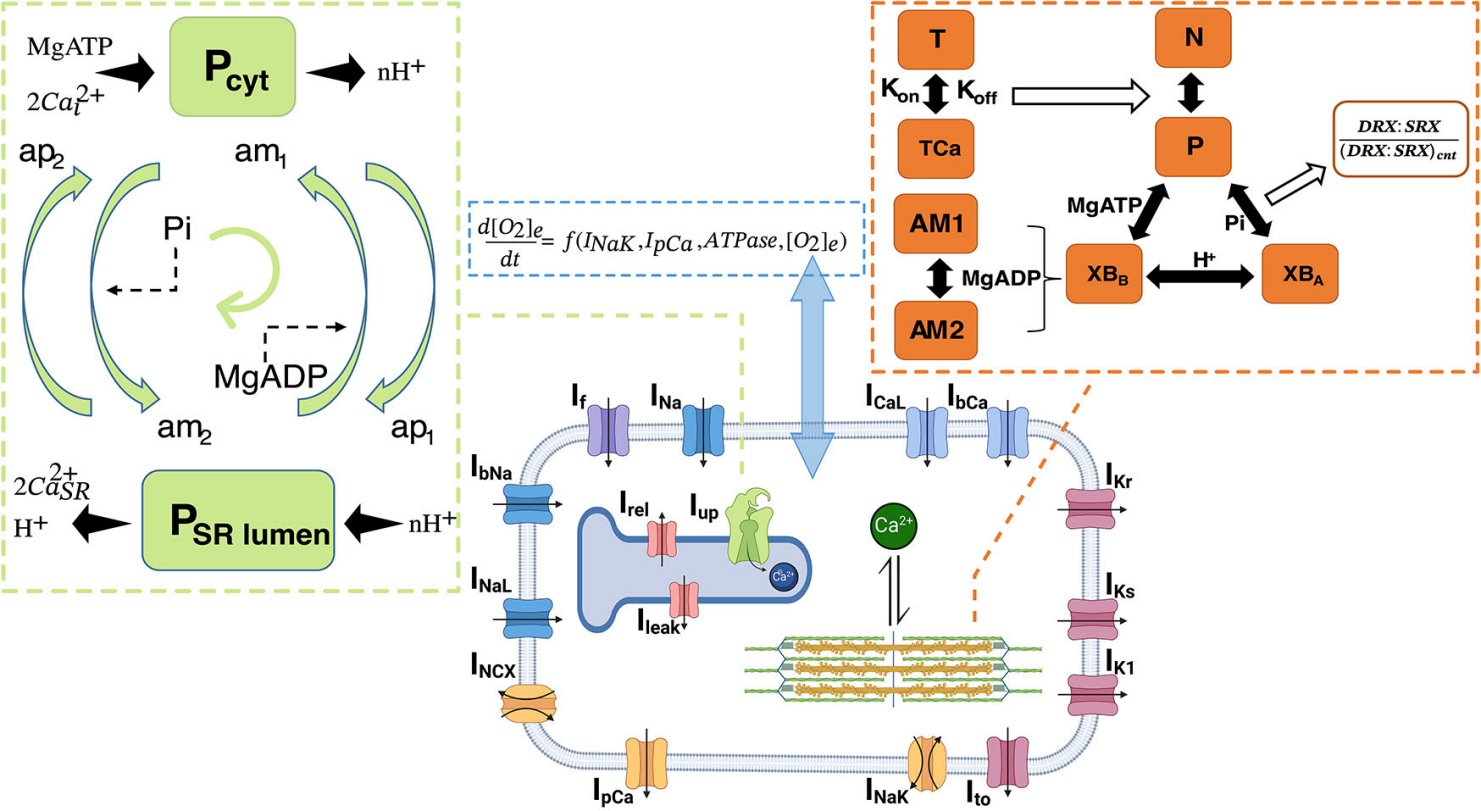
**Schematic of the hiMCES model.** Left: a two-state SERCA model capturing the binding/unbinding of ADP and inorganic phosphate (Pi). The apparent rate constants ap_1_, ap_2_, am_1_ and am_2_ depend on cytosolic and SR Ca^2+^ concentrations, protons and SERCA dissociation constants. Center: schematic of hiMCES main cell compartments, ionic currents and the model extracellular O_2_ dynamics. The middle panel was created with BioRender.com. Right: schematic of the myofilament crossbridge (XB) cycling of hiMCES model. [O_2_]_e_, extracellular oxygen concentration; AM1 and AM2, two closely related substates that contribute equally to the creation of tension (Tran et al., 2010); *ATPase*, contractile ATPase rate; Ca_SR_, sarcoplasmic reticulum Ca^2+^ concentrations; cnt, control; DRX, disordered relaxed myosin state; I_bCa_, background Ca^2+^ current; I_bNa_, background Na^+^ current; I_CaL_, L-type Ca^2+^ current; I_f_, funny current; I_K1_, inward rectifier current; I_Kr_, rapid delayed rectifier K^+^ current; I_Ks_, slow delayed rectifier K^+^ current; I_leak_, leakage current; I_Na_, fast Na^+^ current; I_NaK_, Na^+^-K^+^ pump current; I_NaL_, late Na^+^ current; I_NCX_, late Na^+^-Ca^2+^ exchanger current; I_pCa_, sarcolemmal Ca^2+^ ATPase pump current; I_rel_, RyR-sensitive release current; I_to_, transient outward K^+^ current; I_up_, SERCA pump flux; N, the off state barring XB formation; nH^+^, stoichiometry parameter for H^+^ binding involved in Ca^2+^/H^+^ counter transport; P, permissive state for XB formation; P_cyt_, lumped states of MgATP, H^+^ and Ca^2+^ binding in the cytosol; P_SR lumen_, lumped states of MgADP, H^+^ and Ca^2+^ binding in the SR (Tran et al., 2009); SRX, super relaxed myosin state; T, troponin; TCa, Ca^2+^-bound troponin; XB_A_, strongly bound XBs prior to myosin rotation; XB_B_, XBs in strongly bound state post myosin rotation.

Fig. 6 shows schematics of the hiMCES model including the key state transitions of the CE and SERCA. The SERCA pump is a P-type ATPase functioning in skeletal (SERCA1a) and cardiac (SERCA2a) muscles (Tran et al., 2009). The details of equations describing ionic and contractile components of hiMCES have been discussed in depth previously (Forouzandehmehr et al., 2021b, 2022; Paci et al., 2018). Here, we detail the mathematical model of the SERCA pump adopted in the hiMCES model as follows.

Considering rapid equilibrium assumptions applicable to physiological and ischemic conditions, Tran et al. (2009) developed a lumped two-state thermodynamic model of SERCA based on the E1-E2 model of Ca^2+^ transport in SERCA (Makinose, 1973; Meis and Vianna, 1979), adding proton (H^+^)-binding kinetics to capture pH-dependence phenomena.

The apparent forward rates (see Fig. 6) are defined as:
(1)

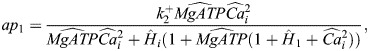

(2)

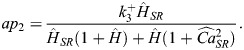


The apparent backward rate parameters are calculated as:
(3)

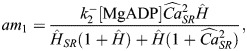

(4)

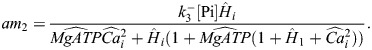


Here,
(5)

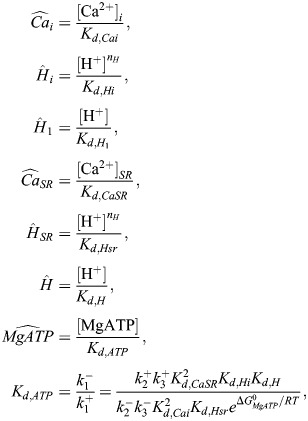
where Pi is inorganic phosphate; [Ca^2+^]_i_ and [Ca^2+^]_SR_ are Ca^2+^ concentrations in the cytosol and SR, respectively; K_d_ is the dissociation constant; *n_H_* is the stoichiometry parameter for H^+^ binding involved in Ca^2+^/H^+^ counter transport; 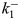, 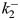 and 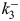 are first-order backward rate constants; 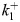, 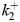 and 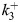 are first-order forward rate constants; and 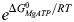
represents the free energy of ATP hydrolysis.

The values of the constants are given in Table 1. The cycling SERCA pump rate (s^−1^) is defined as:
(6)

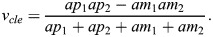
Finally, following Liu et al. (2016, 2019), SERCA pump flux, I_up_, is given by multiplying v_cle_ by a scale factor, S (Eq. 7), which is equal to the ratio of maximum I_up_ obtained by the original formulation in the hiMCE model and maximum v_cle_ in the control condition. Correspondingly, Eq. 8 gives the proportion of the phosphorylated states (PSP).
(7)

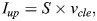

(8)

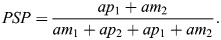


### The model of ischemia

Following an established approach (Dutta et al., 2017; Ledezma et al., 2019; Rodriguez et al., 2006; Weiss et al., 2009), the ischemic severity was divided into two stages, SEV1 and SEV2, representing a mild (onset to 5 min post insult) and a severe (10-12 min post-ischemic insult) model, respectively. We simulated the ischemia at these two severity levels using the data given in Table 1. Hyperkalemia was simulated solely by increasing extracellular potassium K_o_ concentration to 6.25 and 9 mM, consistently with the approach in Kazbanov et al. (2014) and Ledezma et al. (2019). Acidosis was modeled by decreasing the maximum conductances of the fast Na^+^ current (I_Na_) and the L-type Ca^2+^ current (I_CaL_) by 12.5% and 25% following Kazbanov et al. (2014) and Ledezma et al. (2019). In a similar fashion, we also decreased the late Na^+^ current (I_NaL_). An ATP-sensitive K^+^ current (I_KATP_) was introduced to the hiMCES model based on a formulation by Kazbanov et al. (2014). The maximum I_KATP_ conductance was rescaled by f_KATP_ values in the control, SEV1 and SEV2 conditions (Table 1). Values of f_KATP_ were calculated based on an ATP- and ADP-dependent approach by Ferrero et al. (1996). In this approach, f_KATP_ is a function of intracellular MgATP and MgADP as follows:
(9)

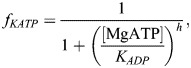
where
(10)

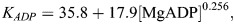

(11)

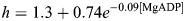
Values of f_KATP_ were obtained using MgATP and MgADP concentrations in different modes (Table 1).

Finally, we introduced a model of oxygen transport connecting [O_2_]_e_ to cardiomyocyte ATP consumption based on approaches in Wan Ab Naim et al. (2021b) and Wei et al. (2014). As oxygen transport in the myocardium is a diffusion phenomenon, the oxygen deprivation is modelled by reducing the oxygen source concentration [O_2_]_s_ to simulate the extracellular oxygen concentration [O_2_]_e_ (Wan Ab Naim et al., 2021a). The oxygen formulation links two levels of O_2_ concentration: one is the O_2_ concentration from the nearby capillary, [O_2_]_s_ (also known as source, supply or bath), and one is the direct extracellular space surrounding the single cell, [O_2_]_e_ (Wan Ab Naim et al., 2021b) (Eq. 12). We also considered the contraction energetics by adding the myofilament ATPase rate (in mM/s) to the oxygen dynamics simulations, following the significant role of contraction in myocardial oxygen consumption reported in ischemia (Bo et al., 2021; Laslett et al., 1985; McDougal and Dewey, 2017). Here, the time rate of change in [O_2_]_e_ is given by:
(12)

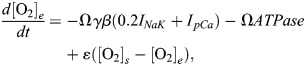
where, Ω was set to 1.6, as the amount of oxygen required to generate 1 mM/s ATP is equal to 1.6 mM/s as per the complete oxidation of ATP (Wei et al., 2014). *γ* is a charge utilization factor converting A/F to mM/s, which here is defined as:
(13)

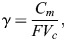
where C_m_=98.7109×10^−12^ F is cell capacitance, F= 96,485.3415 C/mol, and V_c_=8800×10^−18^ m^3^ is cell volume. *ATPase* denotes contractile ATPase rate (mM/s). We set *β*=0.2658 following the experimental rat heart data indicating that contraction contributes to 79% and cell maintenance to 21% of cardiomyocyte energy consumption (McDougal and Dewey, 2017; Rolfe and Brown, 1997). The oxygen diffusion rate ε=5 s^−1^ was calculated based on Fick's law (Cressman et al., 2009; Endeward, 2012):
(14)

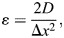
where D=2.5×10^−10^ m^2^/s is the O_2_ diffusion coefficient in the IR model of rat cardiomyocytes (Uchida et al., 1992), and Δ*x*=10 µm is the average distance of cardiomyocytes for intact human cardiac tissues (Endeward, 2012). The baseline of source (bath) oxygen concentration, [O_2_]_s_, was set to [O_2_]_e_=0.133 mM (McDougal and Dewey, 2017).

**Fig. 7.**
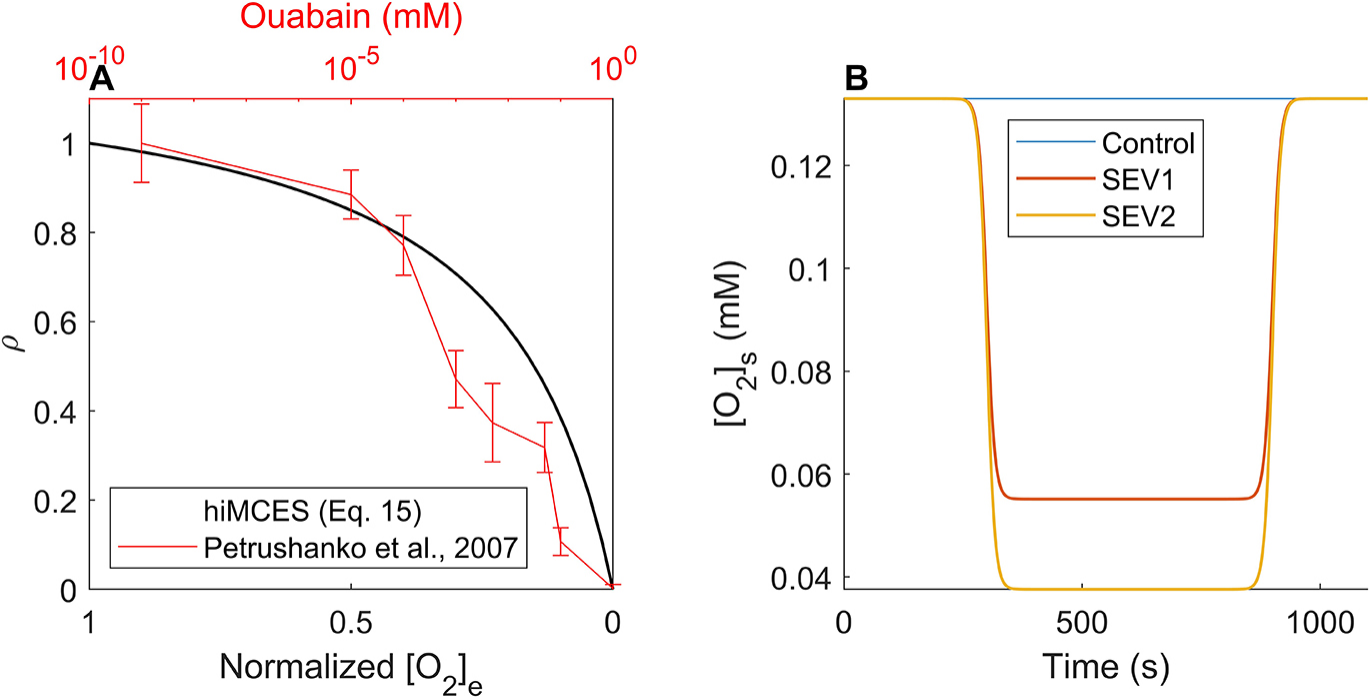
**The oxygen dynamics model links the capillary level [O_2_]_s_ to extracellular [O_2_]_e_ concentration, contractile ATPase and ionic blockades in ischemia.** (A) *ρ* function maps the I_NaK_ and I_pCa_ inhibition to oxygen deprivation and vice versa consistent with ouabain-induced inhibition on I_NaK_ activity. (B) The temporal change in [O_2_]_s_ for different severities of ischemia SEV1 and SEV2 is shown. *ρ* denotes normalized I_NaK_ activity.

Oxygen dynamics affect Na^+^/K^+^ ATPase pump (I_NaK_) and sarcolemmal Ca^2+^ pump (I_pCa_) through setting f_NaK_ and f_pCa_ equal to a sigmoid function *ρ* (Petrushanko et al., 2007), which is then multiplied by the maximum conductance of the currents (Wan Ab Naim et al., 2021b; Wei et al., 2014). In fact, *ρ* is a an O_2_-dependent function that links the effect of oxygen deficiency to I_NaK_ and I_pCa_ and vice versa. It couples the oxygen dynamics to the cell electrophysiology and is given by:
(15)

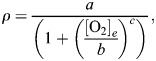
where a=1.215, b=0.215 and c=−1. The ouabain-sensitivity approach has been used as an effective index in studying oxygen dependence in hypoperfusion (Chang et al., 2013; Petrushanko et al., 2007). The values of a, b and c were selected so that *ρ* replicates the oxygen deprivation as an ouabain-like curve reported for I_NaK_ in hypoperfusion (Chang et al., 2013; Sepp et al., 2014) (Fig. 7A). Briefly, a and b were calculated so that the curve IC_50_ would be 15% of the maximum concentration in accord with Ouabain IC_50_=15 nM in a dose-response curve range of 0-100 nM (Butt et al., 2000), and the Hill coefficient would be a negative ouabain Hill coefficient, i.e. c=−1 (Ferrandi et al., 1997). Due to lack of direct measurement data, for SEV1 and SEV2 of ischemia, we calculated [O_2_]_s_ values corresponding to 20% and 31% of I_NaK_ inhibition (Table 1) using the ouabain-like curve (Fig. 7A) mapping oxygen deprivation to I_NaK_ activity (Petrushanko et al., 2007). Fig. 7B shows the temporal change in [O_2_]_s_ in ischemia-reperfusion simulations.

### The model of ischemia reperfusion

As a step toward capturing IR-induced arrhythmia in the IR simulations, first, we took the approach taken previously to simulate EADs (Forouzandehmehr et al., 2021b). We tuned the maximum conductances of the hiMCES model with a coefficient SET that had generated EADs in the Paci2020 electrophysiology model (Paci et al., 2021). The Paci2020 (Paci et al., 2020) and the hiPSC-CM-CE (Forouzandehmehr et al., 2021b) models had predicted EADs in response to 95% of rapid delayed rectifier K^+^ current (I_Kr_) inhibition. Here, for the IR simulations only, during the ischemic phase, at 350-850 s (Fig. 7B), we inhibited two major contributors to the repolarization reserve, I_Kr_ and slow delayed rectifier K^+^ current (I_Ks_), by 85% consistently with IR experimental (Chen et al., 2017; Yin et al., 2017) and ischemic computational (Dutta et al., 2017) reports. For the IR simulations, we used SEV1 values of ischemia (Table 1). All parameters transitions from the physoxia (0-350 s) to the ischemic (350-850 s) phase and returning to physoxia (*in vivo* O_2_ level of blood vessels calculated in McDougal and Dewey, 2017) (850-1100 s) were simulated by an ouabain-like behavior of inhibition (Fig. 7A) to obtain more realistic results specifically at the onset of ischemia and reperfusion phases. For example, instead of instantly switching K_o_ from 5 to 6.25 mM, it took a transition like [O_2_]_s_ at SEV1 (Fig. 7B).

### Model implementation and standard simulation protocol

The hiMCES model consists of 33 ordinary differential equations, and we used MATLAB ode15s as the integrator. To obtain the steady state, the results were reported after 800 s of simulation run time. In Fig. 7B, the model is run with the solution of the last second of an 800-s run, i.e. the control results are already in steady state. Also, as seen in Fig. 7B, the 500 s of model running in ischemic conditions is to ensure a steady state after transition to SEV1/SEV2. In fact, the SEV1 and SEV2 parameters represent the two severities of acute ischemia. Thus, in computational simulations, the run time (shown in Fig. 7B) is to ensure a steady state and does not have to match the time protocols of ischemic *in vitro* experiments. Furthermore, instead of an instantaneous switch from normal to ischemic conditions and vice versa, toward a more realistic modeling, we simulated the shift from normal to ischemic phase and vice versa as an ouabain-like transition to achieve more realistic findings, especially during the onset of the ischemia and reperfusion phases. All the results are in spontaneous beating condition unless mentioned otherwise. Starting from a steady-state solution in the control condition, the model was run for 250 s, and the parameters were adopted to the ischemic mode in the switch phase from 250 to 350 s. Next, the full ischemic phase was run for 500 s (from 350 to 850 s), the transition (onset of reperfusion) starts from 850 to 950 s, and finally, the model was run for 200 s following the last transition (Fig. 7B). The IR results (Fig. S13, Figs 4 and 5) were obtained by the hiMCES version with tuned maximum conductances and currents (coefficient SET).

## Supplementary Material

10.1242/dmm.050365_sup1 Supplementary information
