# Supplementary material for: In silico study of the mechanisms of hypoxia and contractile dysfunction during ischemia and reperfusion of hiPSC cardiomyocytes
Source: Dis Model Mech. 2024 Apr 26;17(4):dmm050365. doi: 10.1242/dmm.050365 (PMC11073514; doi:10.1242/dmm.050365)
Supplement: Supplementary information [file dmm-17-050365-s1.pdf]

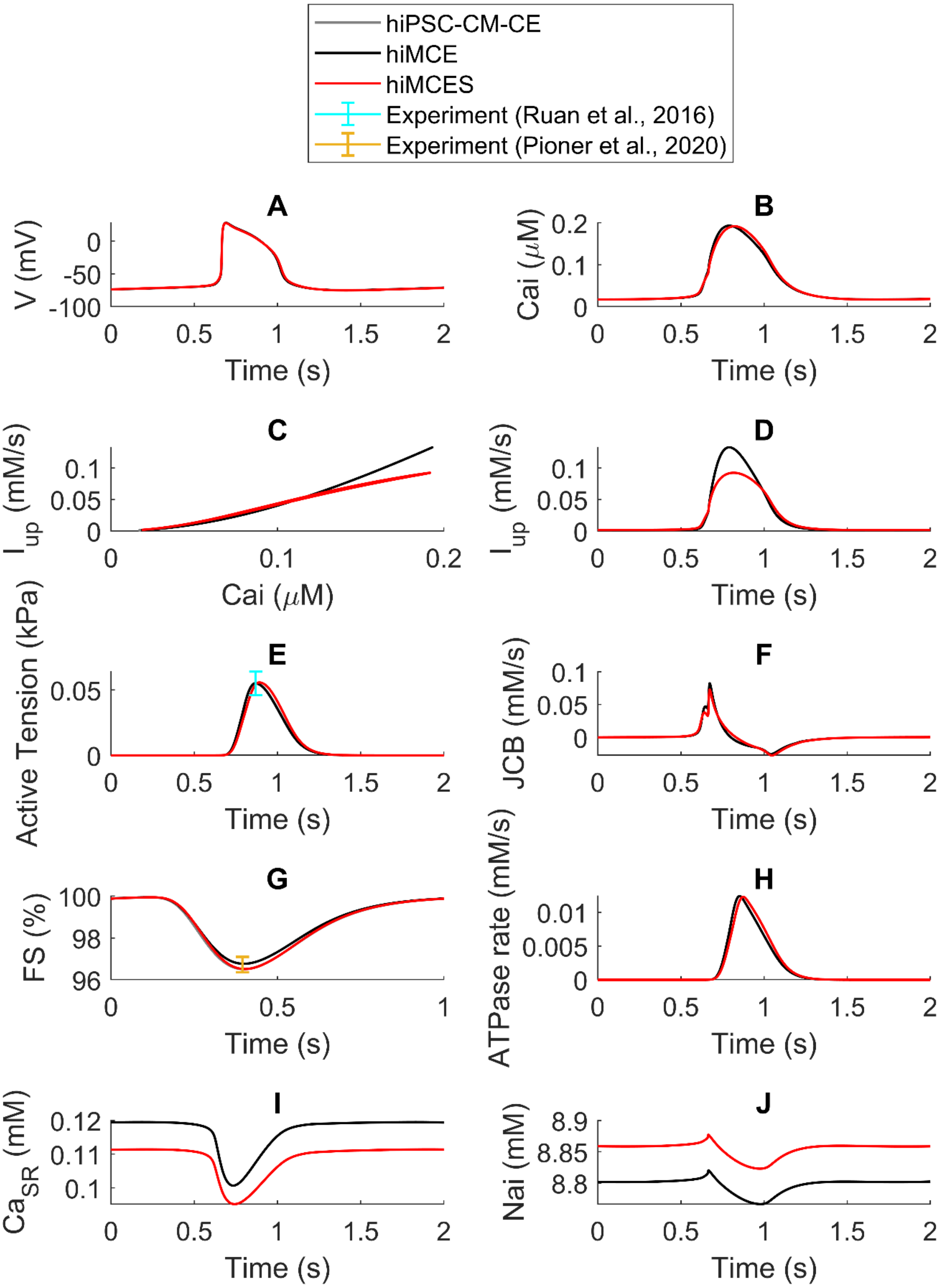

Fig. S1. Standard outcomes of the hiMCES model in control condition compared to hiPSC-CM-CE (Forouzandehmehr et al., 2021) and hiMCE (Forouzandehmehr et al., 2022) models. Action Potentials (A), Ca^{2+} Transients (B), SERCA uptake (I_{up}) (C&D), active tensions (E), flux of Ca^{2+} toward the contractile element (F), fractional cell shortening (G), contractile ATPase rate (H), sarcoplasmic reticulum Ca^{2+} concentration (I), and intracellular Na^+ concentrations (J). Panel G data are at 1 Hz pacing.

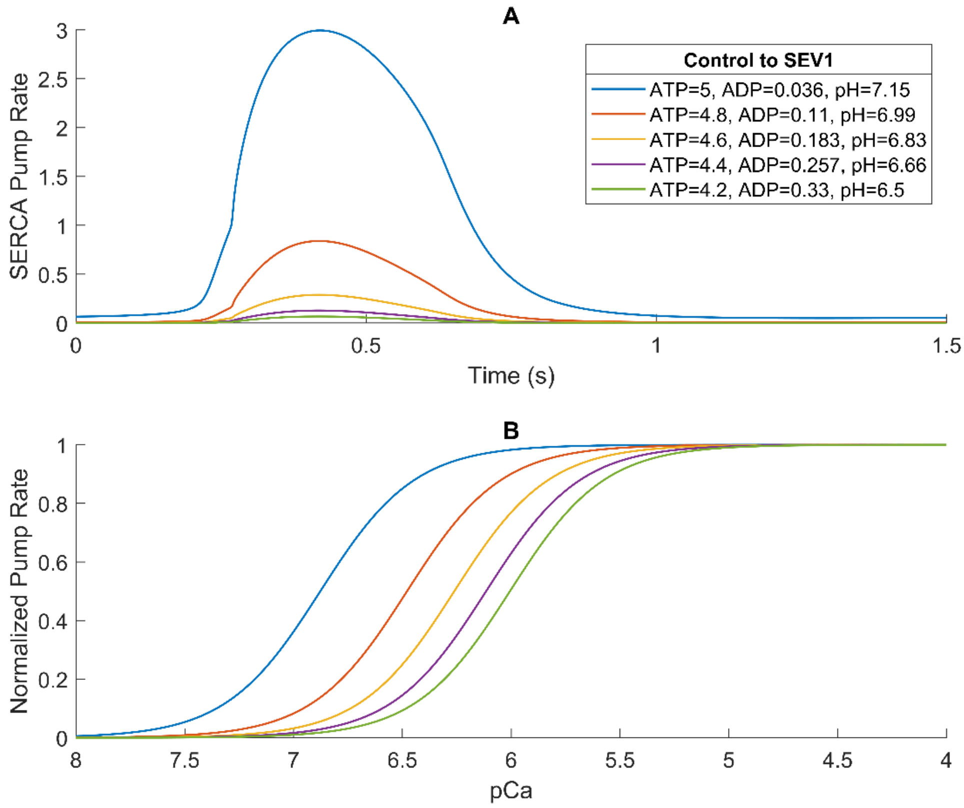

Fig. S2. The effect of metabolite changes on the SERCA model behavior. SERCA pump rate (A) and Ca^{2+} sensitivity (B).

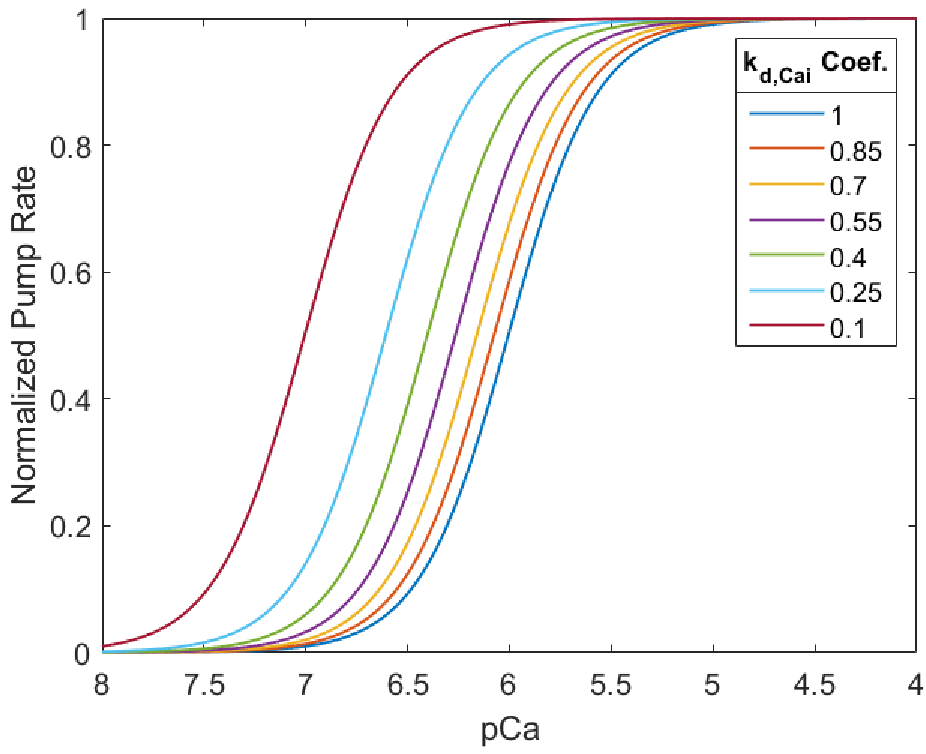

Fig. S3. Ca^{2+} sensitivity of SERCA pump rate in response to different Kd,Cai coefficients.

Disease Models & Mechanisms • Supplementary information

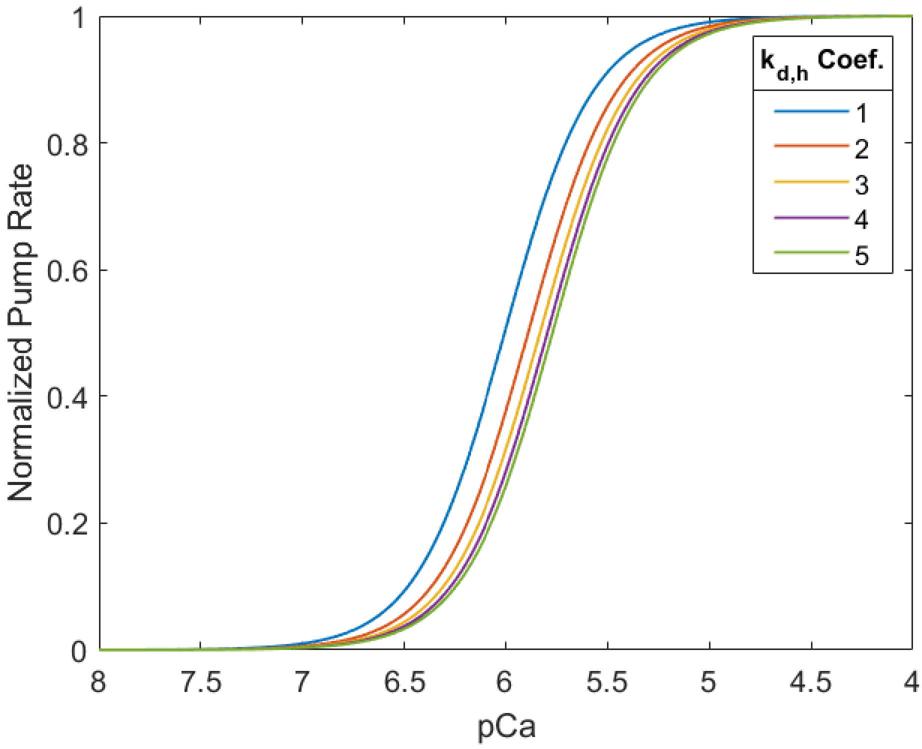

Fig. S4. Ca^{2+} sensitivity of SERCA pump rate in response to different Kd,h coefficients.

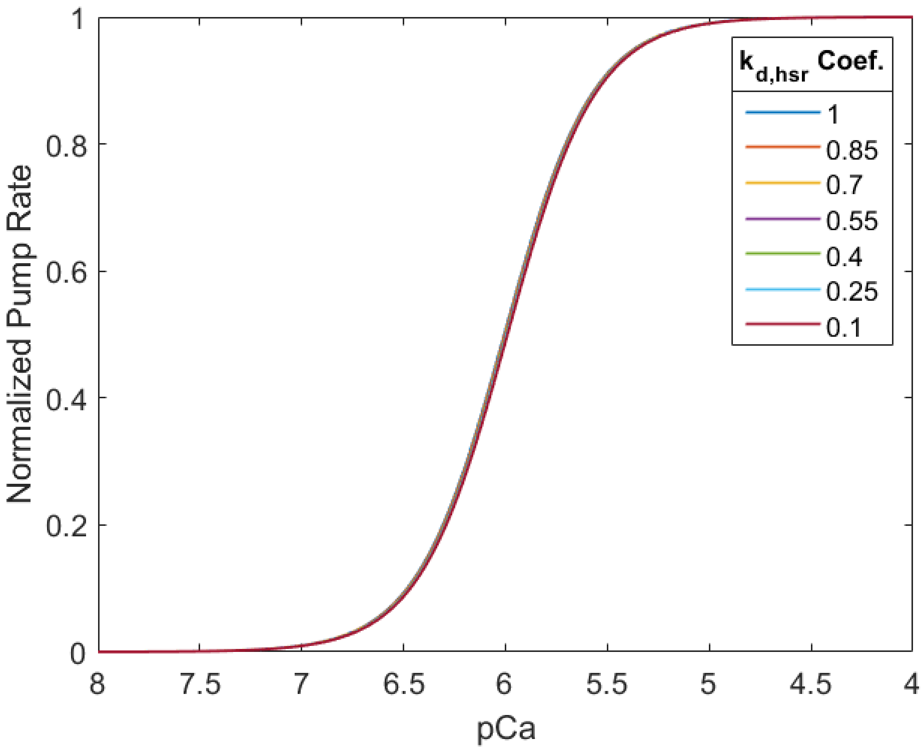

Fig. S5. Ca^{2+} sensitivity of SERCA pump rate in response to different Kd,Hsr coefficients.

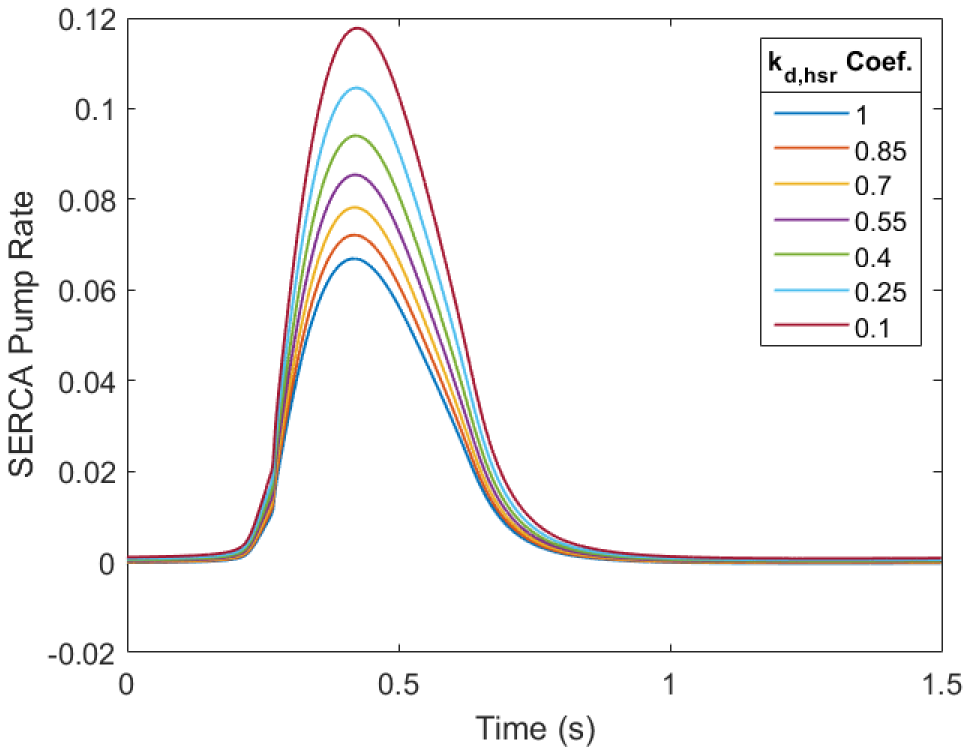

Fig. S6. SERCA pump amplitude vs time in response to different Kd,hsr coefficients.

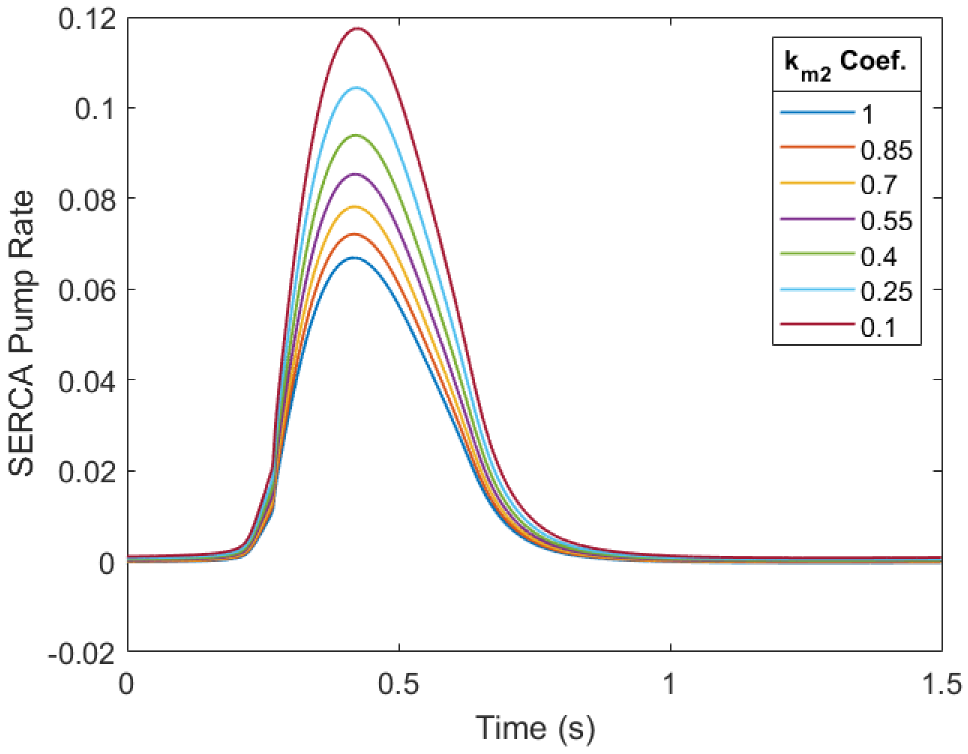

Fig. S7. SERCA pump amplitude vs time in response to different km2 coefficients.

Disease Models & Mechanisms • Supplementary information

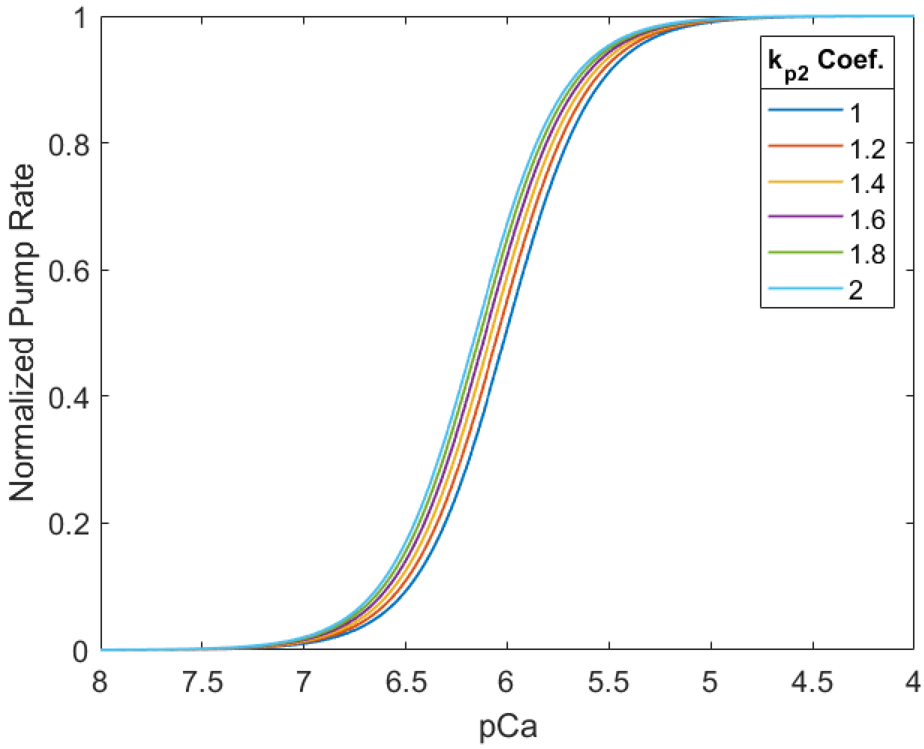

Fig. S8. Ca^{2+} sensitivity of SERCA pump rate in response to different kp2 coefficients.

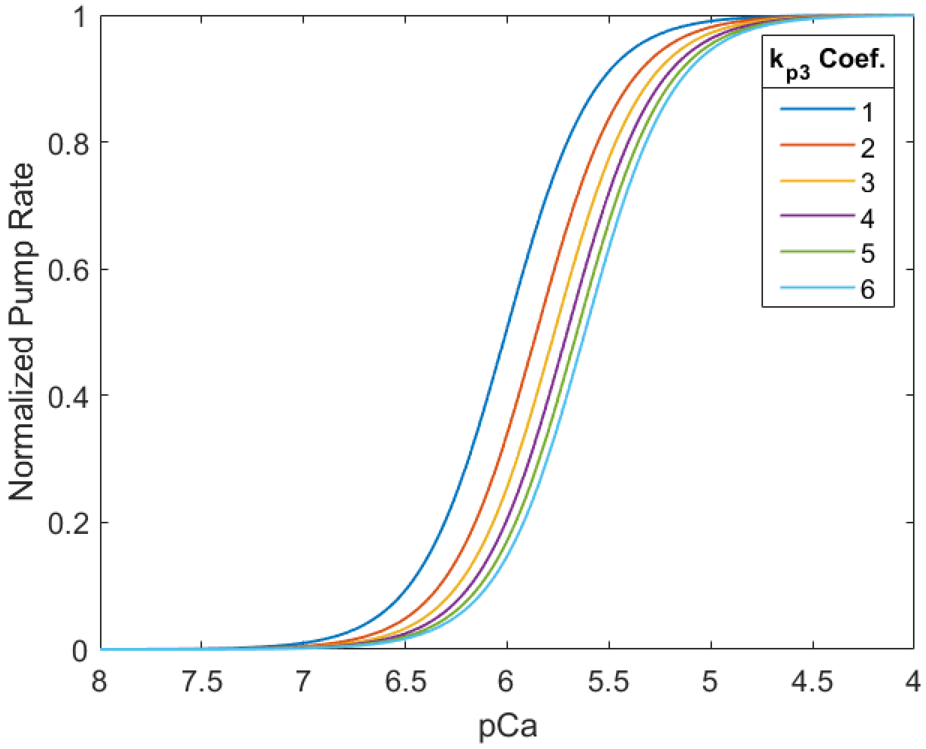

Fig. S9. Ca^{2+} sensitivity of SERCA pump rate in response to different kp3 coefficients.

Disease Models & Mechanisms • Supplementary information

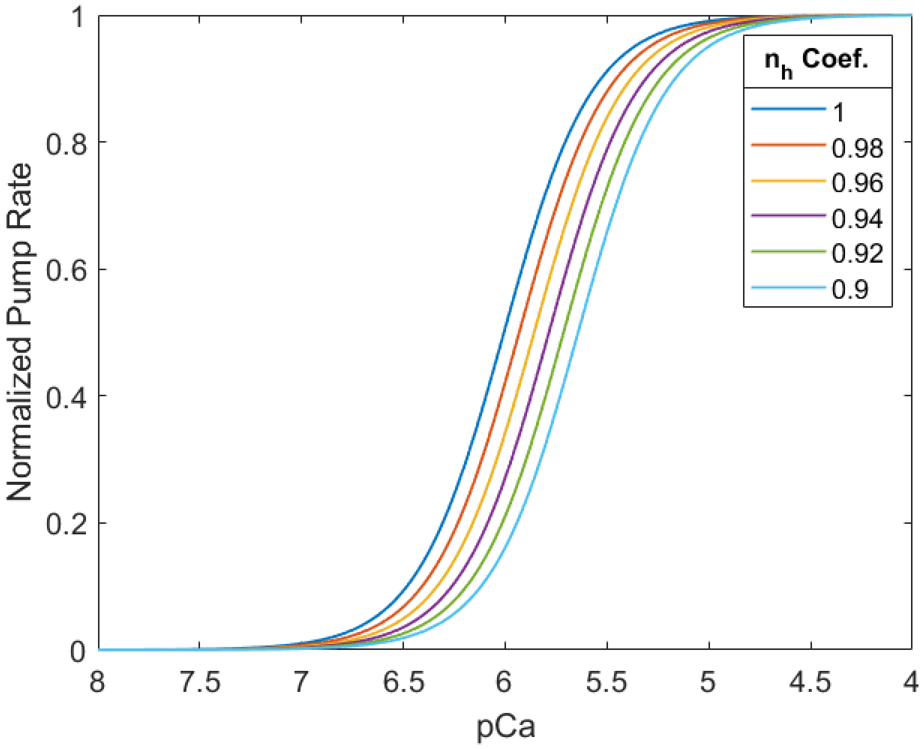

Fig. S10. Ca^{2+} sensitivity of SERCA pump rate in response to different n_h coefficients.

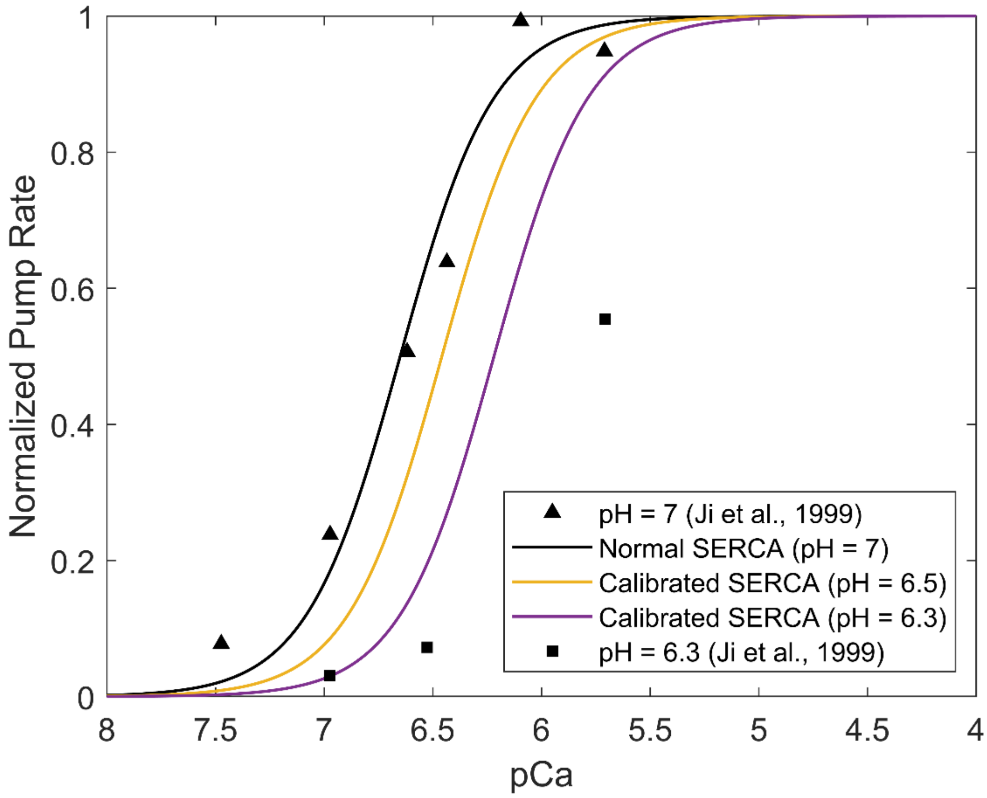

Fig. S11. Ca^{2+} sensitivity of normal and calibrated SERCA pump rates at different pH levels.

Disease Models & Mechanisms • Supplementary information

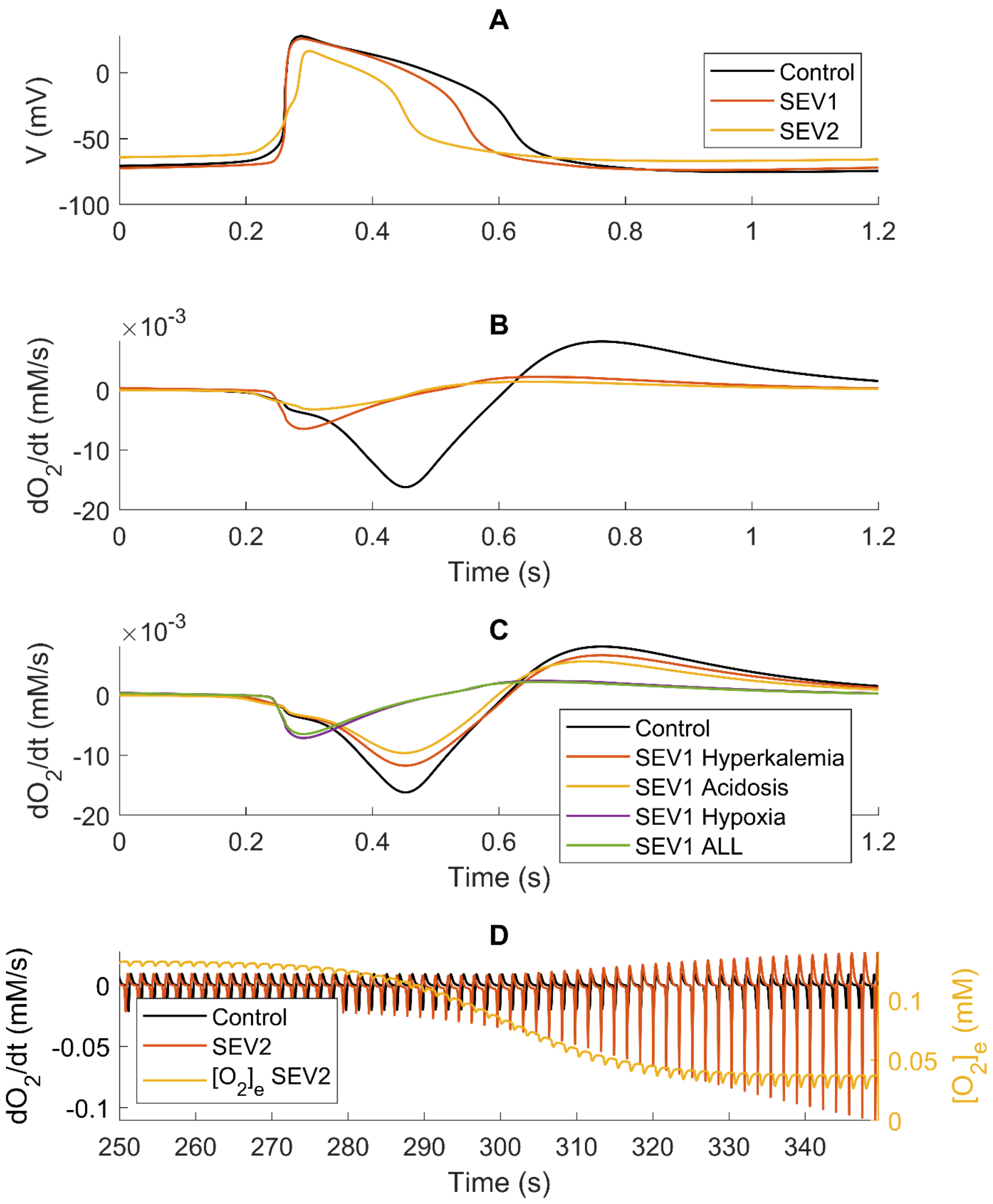

Fig. S12. The model simulates effects of the ischemic insult in total and separated by mechanisms on oxygen consumption rate. Simulated action potentials (A) and oxygen dynamics in control and ischemic conditions: severities 1 and 2 (B), the effect of separate mechanisms of ischemia on oxygen consumption rate (C), and time-dependent behavior of oxygen consumption rate at severity 2 at the onset of ischemia (D).

Disease Models & Mechanisms • Supplementary information

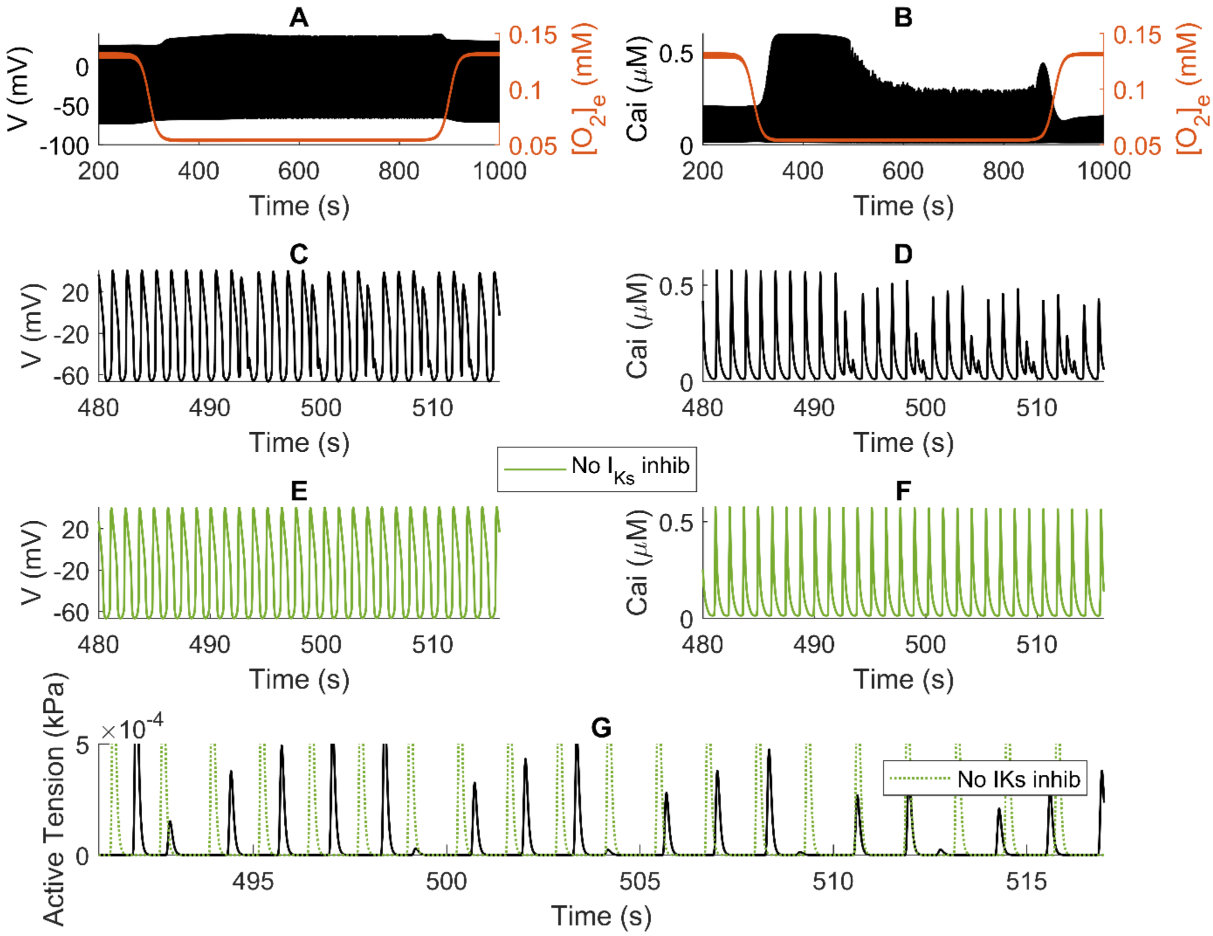

Fig. S13. I_{Ks} activation significantly diminishes ischemia-induced abnormalities. The Ischemia-reperfusion model of hiMCES (A&B) could capture the ischemic-induced abnormalities and simulate the cardioprotective effect of activation of slow delayed rectified K^+ current (I_{Ks}) in action potential (AP) (C&E), Ca^{2+} transients (CaTs) (D&F), and active tension (G).

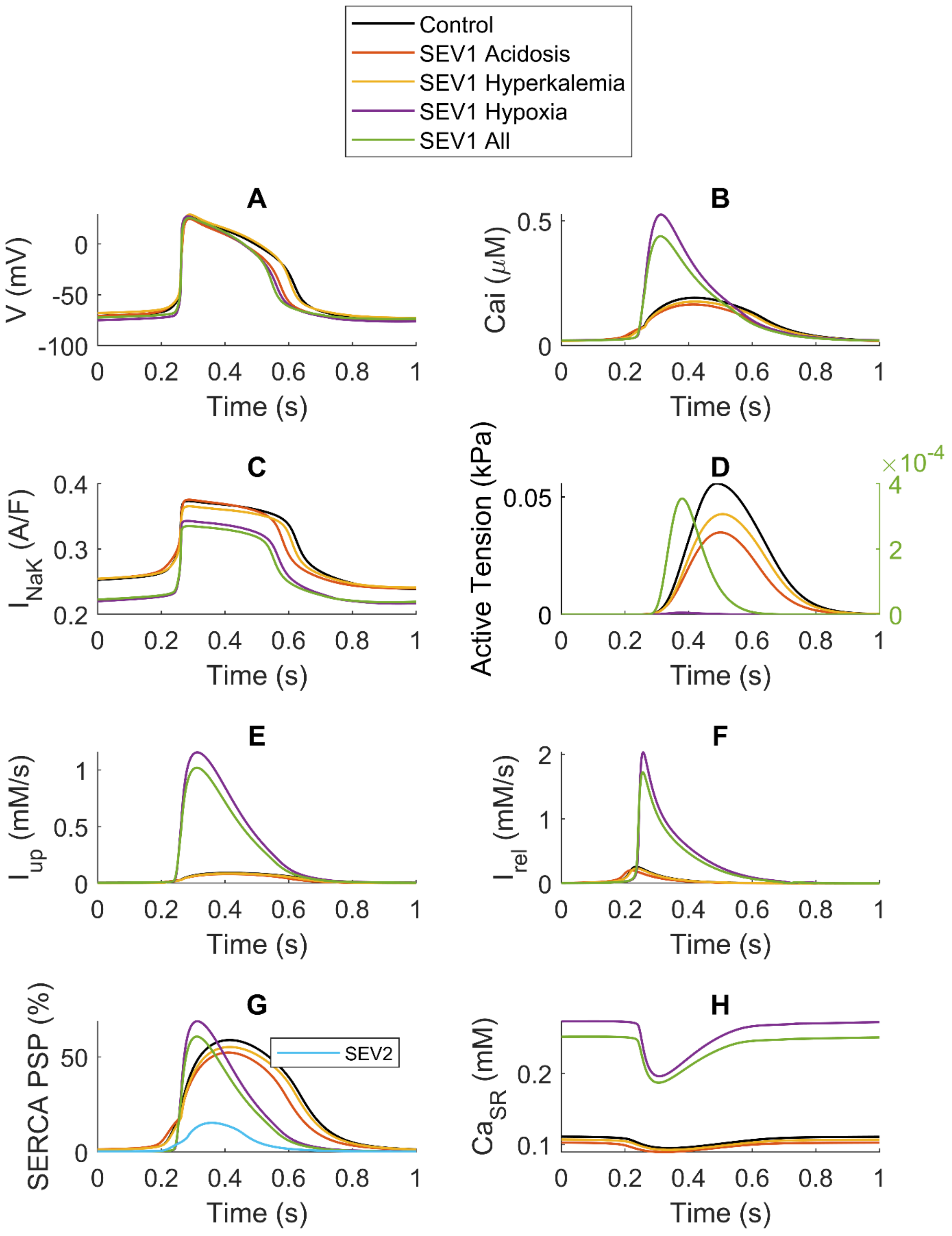

Fig. S14. Response of hiMCES model to mechanisms of ischemia. Action potentials (A), calcium transients (B), Na^+/K^+ pump (C), active tensions (D), SERCA uptake pump (E), SERCA release current (F), SERCA phosphorylation (G), Sarcoplasmic Reticulum Ca^{2+} concentration (H).

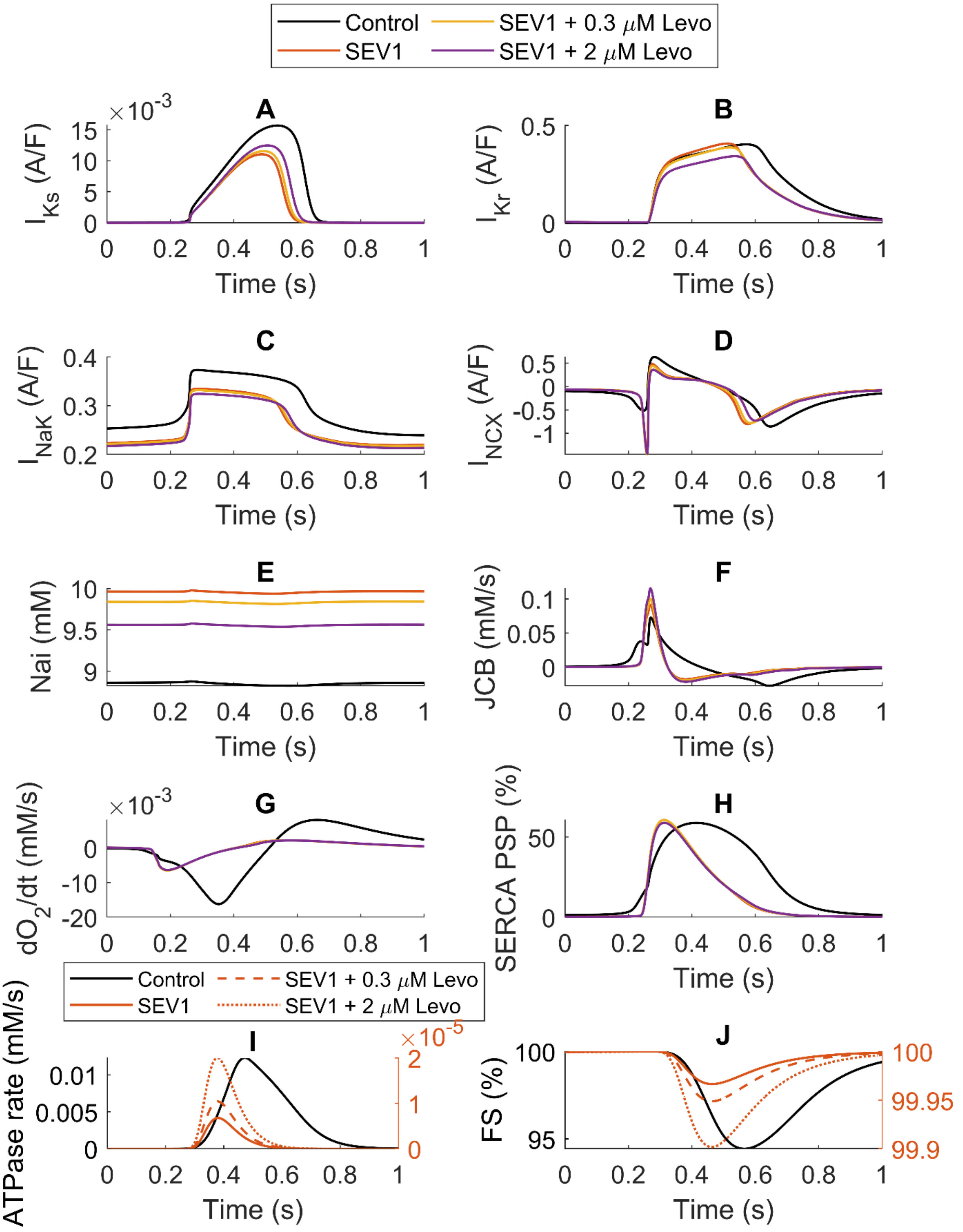

Fig. S15. Response of hiMCES model to Levosimendan effect on ischemia. Slow delayed rectified K^+ currents (A), rapid delayed rectified K^+ currents (B), Na^+/K^+ pumps (C), Na^+-Ca^{2+} exchangers (D), intracellular Na^+ concentrations (E), Ca^{2+} flux toward myofilament (F), oxygen consumption rates (G), SERCA phosphorylations (H), contractile ATPase rate (I), and fractional shortening (J).

Disease Models & Mechanisms • Supplementary information

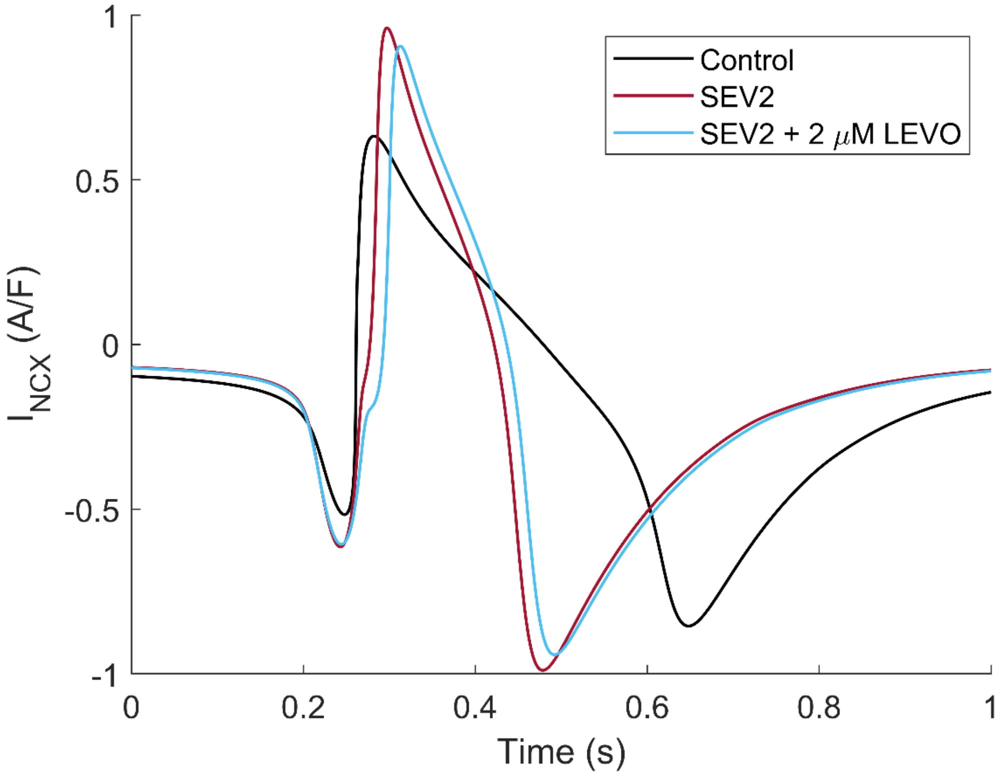

Fig. S16. Simulated I_{NCX} traces at SEV2 of ischemia and effect of 2 μM Levosimendan (LEVO).

Disease Models & Mechanisms • Supplementary information

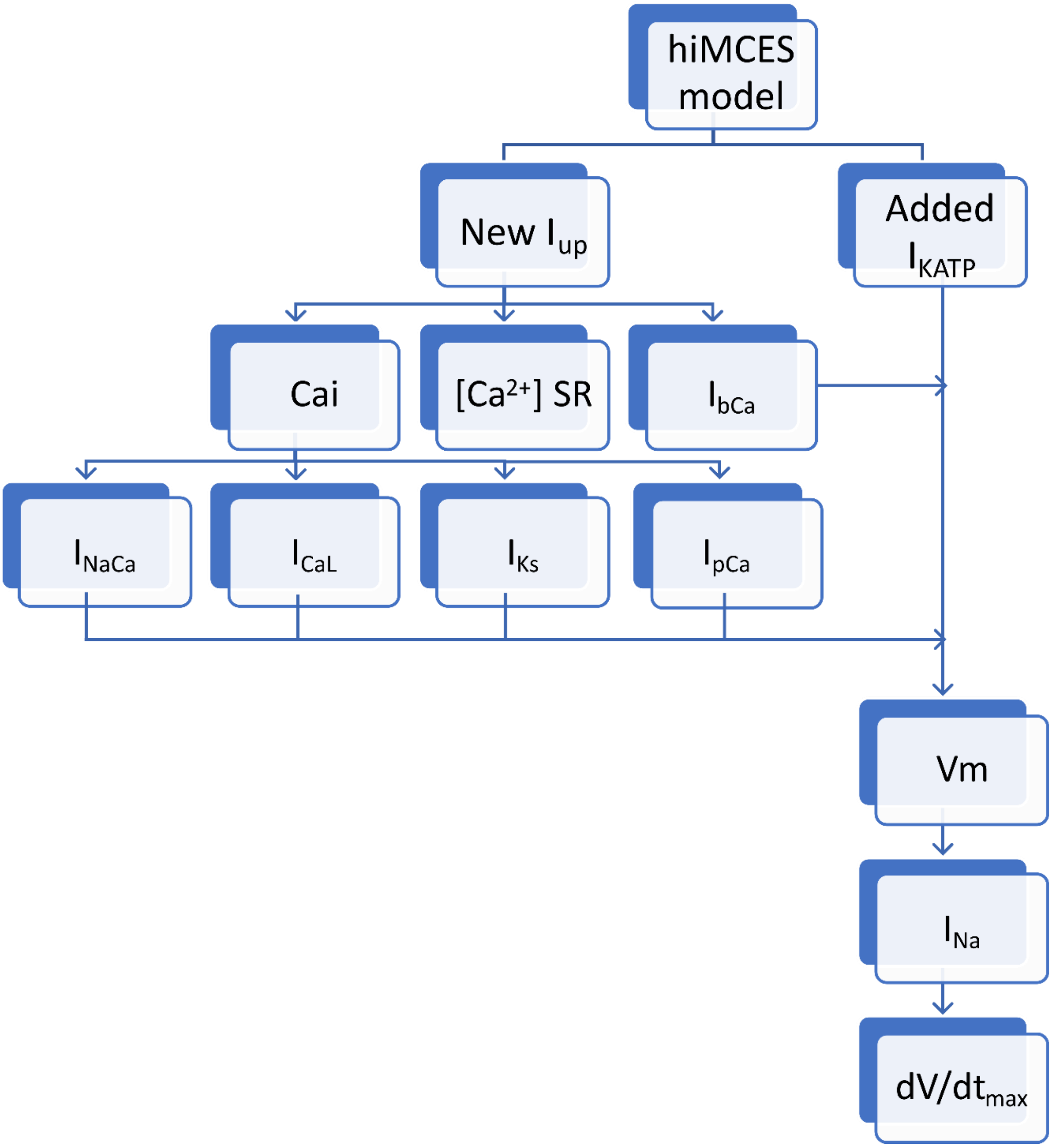

Fig. S17. Factors influencing the calculation of fast Na^+ current (I_{Na}) and maximum upstroke velocity (dV/dt_{max}).

Table S1. Action potential (AP), Ca^{2+} transients (CaT), and biomechanical biomarkers of hiMCES model and three corresponding hiPSC-CM in silico models computed in spontaneous condition in comparison with the experimental values (Paci et al., 2018; Paci et al., 2020). APA: AP amplitude, MDP: maximum diastolic potential, CL: cycle length, dV/dt max: maximum upstroke velocity, APD_{10} and APD_{30} and APD_{90}: AP duration at 10, 30, 90% of repolarization, respectively, AP Tri: AP triangulation index. The simulated biomarkers of CaT are DURATION: duration of the transient, $tRise_{10, peak}$: time to peak, $tRise_{10, 50}$ and $tRise_{10, 90}$: rise time from 10 to 50% and 90% of maximum threshold, respectively, and $tDecay_{90,10}$: decay time from 90 to 10%. AT: Active tension, RT_{50}: time from peak contraction to 50% of relaxation, %FS: percent of fractional shortening. The experimental ranges for contraction biomarkers were imported from (Forouzandehmehr et al., 2021).

No.	Biomarker	Paci2020, (Paci et al., 2020)	hiPSC-CM-CE, (Forouzandehmehr et al., 2021)	hiMCE, (Forouzandehmehr et al., 2022)	hiMCES	Exp. Value (Mean±SD), (Paci et al., 2018; Paci et al., 2020)
1	APA (mV)	102	103	103	103	104±6
2	MDP (mV)	-74.9	-75.0	-75.0	-75.2	-75.6±6.6
3	AP CL (ms)	1712	1644	1644	1694	1700±548
4	dV/dt max (V/s)	20.5	23.9	24.0	10.7	27.8±26.3
5	APD_{10} (ms)	87.0	95.0	95.1	91.6	74.1±26.3
6	APD_{30} (ms)	224	238	238	238	180±59
7	APD_{90} (ms)	390	403	403	413	415±119
8	AP Tri	2.8	2.9	3	2.9	2.5±1.1
9	CaT DURATION (ms)	691	693	693	693	805±188
10	CaT $tRise_{10, peak}$ (ms)	184	163	163	197	270±108
11	CaT $tRise_{10,50}$ (ms)	54.9	46.2	45.9	54	82.9±50.5
12	CaT $tRise_{10,90}$ (ms)	118	102	102	125	167±70
13	CaT $tDecay_{90,10}$ (ms)	341	343	343	317	410±100
14	AT magnitude (kPa)	-	0.055	0.055	0.0557	0.055±0.009
15	RT_{50} (ms)	-	161	158	154	158±12.1
16	%FS	-	3.45	3.23	3.43	3.27±0.37

Table S2. Standard and reparametrized SERCA model parameters. The second column was taken directly from (Tran et al., 2009).

Parameter	Control	Ischemia (SEV1 & SEV2)
k_1^+ (mM^{-1} s^{-1})	25900	2 × 25900
k_2^+ (s^{-1})	2540	2 × 2540
k_3^+ (s^{-1})	20.5	5 × 20.5
k_1^- (mM^{-1} s^{-1})	2	2
k_2^- (mM^{-1} s^{-1})	67200	0.1 × 67200
k_3^- (mM^{-1} s^{-1})	149	149
$K_{d,Cai}$ (mM)	0.91	0.1 × 0.91
$K_{d,CaSR}$ (mM)	2.24	2.24
$K_{d,H1}$ (mM)	1.09 ×10^{-5}	1.09 ×10^{-5}
$K_{d,Hi}$ (mM^2)	3.54 ×10^{-3}	3.54 ×10^{-3}
$K_{d,Hsr}$ (mM^2)	1.05 ×10^{-8}	0.1 × 1.05 ×10^{-8}
$K_{d,H}$ (mM)	7.24 ×10^{-5}	5 × 7.24 ×10^{-5}
n_H	2	0.97 × 2

Table S3. Parameters used to model the effect of Levosimendan (LEVO). I_{NaF}: fast Na^+ current, I_{CaL}: L-type Ca^{2+} current, I_{Kr}: Rapid delayed rectified K^+ current, K_{on}: rate constant for Ca^{2+} to troponin C binding affinity (Rice et al., 2008; Tran et al., 2010), f_{KATP}: fraction of open I_{KATP} channels (Ferrero et al., 1996; Kazbanov et al., 2014).

LEVO (μM)	I_{NaF} block (%)	I_{CaL} block (%)	I_{Kr} block (%)	K_{on} coefs.	f_{KATP} activation (%)
0.3	0.35	1.04	5.11	1.1	0.2385
2	2.28	6.57	16.38	1.32	11.8538
10	10.45	26.01	36.91	1.88	84.3903

Table S4. Action potential durations at 90% of repolarization (APD90s) simulated and compared with in vitro hiPSC-CMs experiment in control mode at 37℃ (Selli et al., 2023). The AP triangulations were calculated as (APD90-APD30)/APD90. LEVO: Levosimendan.

#	LEVO (μM)	Simulation (control) APD90 (ms)	Simulation (ischemia SEV2) APD90 (ms)	In vitro (Selli et al., 2023) APD90 (ms)
1	0	413	293.3	340.3
2	0.3	419.4	296.7	346.4
3	2	425.8	306.2	353.3
4	% of change (0 to 2 μM)	3.1	4.4	3.8

Disease Models & Mechanisms • Supplementary information

Supplementary references

Ferrero, J. M., Sáiz, J., Ferrero, J. M. and Thakor, N. V. (1996). Simulation of Action Potentials From Metabolically Impaired Cardiac Myocytes. *Circulation Research* **79**, 208–221.

Forouzandehmehr, M., Koivumäki, J. T., Hyttinen, J. and Paci, M. (2021). A mathematical model of hiPSC cardiomyocytes electromechanics. *Physiological Reports* **9**,.

Forouzandehmehr, M., Paci, M., Koivumäki, J. T. and Hyttinen, J. (2022). Altered contractility in mutation-specific hypertrophic cardiomyopathy: A mechano-energetic in silico study with pharmacological insights. *Frontiers in Physiology* **13**,.

Kazbanov, I. V., Clayton, R. H., Nash, M. P., Bradley, C. P., Paterson, D. J., Hayward, M. P., Taggart, P. and Panfilov, A. V. (2014). Effect of Global Cardiac Ischemia on Human Ventricular Fibrillation: Insights from a Multi-scale Mechanistic Model of the Human Heart. *PLoS Computational Biology* **10**, e1003891.

Paci, M., Pölönen, R. P., Cori, D., Penttinen, K., Aalto-Setälä, K., Severi, S. and Hyttinen, J. (2018). Automatic optimization of an in silico model of human iPSC derived cardiomyocytes recapitulating calcium handling abnormalities. *Frontiers in Physiology*.

Paci, M., Passini, E., Klimas, A., Severi, S., Hyttinen, J., Rodriguez, B. and Entcheva, E. (2020). All-Optical Electrophysiology Refines Populations of In Silico Human iPSC-CMs for Drug Evaluation. *Biophysical Journal*.

Rice, J. J., Wang, F., Bers, D. M. and De Tombe, P. P. (2008). Approximate model of cooperative activation and crossbridge cycling in cardiac muscle using ordinary differential equations. *Biophysical Journal*.

Selli, A. L., Ghasemi, M., Watters, T., Burton, F., Smith, G. and Dietrichs, E. S. (2023). Proarrhythmic changes in human cardiomyocytes during hypothermia by milrinone and isoprenaline, but not levosimendan: an experimental in vitro study. *Scandinavian Journal of Trauma, Resuscitation and Emergency Medicine* **31**, 61.

Tran, K., Smith, N. P., Loiselle, D. S. and Crampin, E. J. (2009). A Thermodynamic Model of the Cardiac Sarcoplasmic/Endoplasmic Ca2+ (SERCA) Pump. *Biophysical Journal* **96**, 2029–2042.

Tran, K., Smith, N. P., Loiselle, D. S. and Crampin, E. J. (2010). A Metabolite-Sensitive, Thermodynamically Constrained Model of Cardiac Cross-Bridge Cycling: Implications for Force Development during Ischemia. *Biophysical Journal* **98**,.
